# Insights into the Genomic and Phenotypic Landscape of the Oleaginous Yeast *Yarrowia lipolytica*

**DOI:** 10.3390/jof9010076

**Published:** 2023-01-04

**Authors:** Frédéric Bigey, Emilie Pasteur, Xymena Połomska, Stéphane Thomas, Anne-Marie Crutz-Le Coq, Hugo Devillers, Cécile Neuvéglise

**Affiliations:** 1 INRAE, Institut Agro, SPO, University Montpellier, 34060 Montpellier, France; 2 Micalis, Université Paris-Saclay, INRAE, AgroParisTech, 78350 Jouy-en-Josas, France; 3 Department of Biotechnology & Food Microbiology, Wroclaw University of Environmental and Life Sciences (WUELS), 50-375 Wroclaw, Poland; 4 IJPB, INRAE, 78000 Versailles, France

**Keywords:** population genomics, phenotype, diversity, killer toxin, transposable elements

## Abstract

Although *Yarrowia lipolytica* is a model yeast for the study of lipid metabolism, its diversity is poorly known, as studies generally consider only a few standard laboratory strains. To extend our knowledge of this biotechnological workhorse, we investigated the genomic and phenotypic diversity of 56 natural isolates. *Y. lipolytica* is classified into five clades with no correlation between clade membership and geographic or ecological origin. A low genetic diversity (π = 0.0017) and a pan-genome (6528 genes) barely different from the core genome (6315 genes) suggest *Y. lipolytica* is a recently evolving species. Large segmental duplications were detected, totaling 892 genes. With three new LTR-retrotransposons of the *Gypsy* family (Tyl4, Tyl9, and Tyl10), the transposable element content of genomes appeared diversified but still low (from 0.36% to 3.62%). We quantified 34 traits with substantial phenotypic diversity, but genome-wide association studies failed to evidence any associations. Instead, we investigated known genes and found four mutational events leading to *XPR2* protease inactivation. Regarding lipid metabolism, most high-impact mutations were found in family-belonging genes, such as *ALK* or *LIP*, and therefore had a low phenotypic impact, suggesting that the huge diversity of lipid synthesis and accumulation is multifactorial or due to complex regulations.

## 1. Introduction

*Yarrowia lipolytica,* an early branching yeast in the *Saccharomycotina subphylum*, has long been considered a model for lipid metabolism. It can grow on a few carbohydrates only but can assimilate many hydrophobic substrates, which makes it a good partner for the bioremediation of oil-polluted environments [1]. From a fundamental perspective, the species is mainly used to better understand lipid uptake, synthesis, degradation, and storage. Indeed, some strains are able to accumulate more than 20% of their dry cell weight as lipids, thus defining them as oleaginous yeasts [2]. From a biotechnological point of view, *Y. lipolytica* is used in a wide range of industrial applications, especially in the oleochemical industry, to synthesize bioplastics and biofuels from low-value substrates generated from industrial or agricultural wastes [3,4,5]. Additional characteristics of this yeast, such as tolerance to inhibitors, growth at high cell density, high protein secretion, safety as well as the availability of synthetic biological tools, make it a workhorse for bioproduction [6]. A comprehensive review of the past and future use of *Y. lipolytica* for white biotechnology applications was recently published [7].

As a model organism, *Y. lipolytica* strains have been fully sequenced and annotated, in particular historical strains and their derivatives (See Table S1 in [7]). In most cases, except, for instance, the type strain CBS 6124 (=ATCC 18942; NRRL YB-423), the natural isolates of *Y. lipolytica* are haploid with either the *MATA* or *MATB* alleles, which facilitates genome assembly [8]. The first sequenced strain, CLIB 122 (=E150), derives from a first cross between the French strain W29 (=CLIB 89; CBS 7504) and the American strain CBS 6124-2 (=CLIB 78), then followed by numerous backcrosses. Its genome sequence and gene annotation were achieved in 2004 by the Génolevures Consortium [9], and their high-quality promoted this genome as a reference. It contains six scaffolds, one per chromosome, totaling 20.5 Mb, with a lower gene density than in other Saccharomycotina, such as *Saccharomyces cerevisiae*, *Candida glabrata*, or *Debaryomyces hansenii* [9], and a higher proportion of intron-containing genes [10]. Ten years after the release of this first genome, the Chinese strain WSH-Z06 was sequenced by Jiangnam University, and in the same year, 2014, the University of Texas at Austin sequenced strain PO1f, which is a lab derivative from W29 [11]. The genome sequence of CLIB 89 (W29), which is one of the two parental strains of E150, was released in 2016 by the University of California, Irvine [12], while in the same year, the Polish strain A101 was sequenced by the Micalis Institute [13]. Among the major lineages used for fundamental research and biotechnological applications, the German strain H222 was sequenced in 2019 [14]. In June 2022, a total of 27 genome sequences were available in public databases, with variable quality of assembly and annotation. However, the last published genome (strain DSM 3286) established a near-contiguous genome sequence from telomere to telomere, with the resolution of the six rDNA clusters in subtelomeric regions [15].

One striking feature of the genomes of this species is the variability of their structure, as reported in an early karyotypic analysis [16] and more recently confirmed by *de novo* genome assemblies in which reciprocal translocations and inversions were detected [12,14]. Chromosomal rearrangements may be induced by repeated sequences such as transposable elements, whose diversity in *Y. lipolytica* is quite high for yeast. LTR-retrotransposons, LINE elements, and DNA transposons of various families have been identified with a variable distribution according to strains [17,18,19,20]. Large families of protein-coding genes are also found in *Y. lipolytica* strains that may contribute to shaping their genomes. Gene family expansion has been particularly observed in lipid metabolism, with 16 lipases, 12 cytochromes P450, 6 acyl-coA oxidases, etc., in strain E150 [21,22,23,24]. A first *Y. lipolytica* pan-genome was established based on the genome of seven strains, using a synteny-dependent Pan-genome Ortholog Clustering Tool approach. The authors found 6042 core orthologues (86.14%) and 972 accessory genes (13.86%), of which 390 consisted of singleton gene models [25]. However, these results may have been biased by the level of completion of certain genomes and the low number of studied strains.

Although *Y. lipolytica* is extensively studied for its metabolic properties, little is known about the genetic and phenotypic diversity of the species. The most studied trait has been the capacity for lipid synthesis and accumulation [2,26]. A few recent studies used other phenotypic screenings to select promising strains for particular applications, some of which tried to correlate phenotypes and genotypes. As an example of phenotypic variation, citric acid production has been assessed in 10 strains and showed a huge variation of production, reaching up to 72.12 g/L for strain K57 in a glucose-based medium in batch culture [27]. In another study where the use of different growth temperatures was evaluated, variable impacts on the growth rate, key metabolites, and lipid accumulation abilities were observed for three strains grown in different media. A comparison of their genotypes revealed a mean polymorphism of three nucleotides per kb, with 10% of them causing non-synonymous mutations in protein-coding genes. Unfortunately, the correlation between phenotypes and genotypes did not lead to compelling results [28]. The only study that compared multiple strains and provided several genomes did not explain which screening was used to select five strains (out of 45) with unique metabolic detoxification capability, nor did it compare their genomes and gene specificities [29].

Here, we analyzed the genomic and phenotypic diversity of 56 haploid strains of *Y. lipolytica*. To this end, we used a collection of strains from widely different geographical and biological origins. Two different approaches were carried out: the first approach combined population genomics and comparative genomics and consisted of searching for Single Nucleotide Polymorphisms (SNPs) and nucleotide insertions or deletions (indels), building a phylogenetic tree, analyzing population structure, and comparing genome architecture and gene content. A phenotyping approach consisting of growth tests on different media and temperatures complemented the genomic approach and allowed us to investigate the genetic basis of some traits.

## 2. Materials and Methods

### 2.1. Strains

We studied 56 wild strains from 22 countries that were collected in different environments such as food (18 strains including 7 strains from dairy products), soil (12 strains), human (10 strains from organs, skin, or clinical samples), industrial products (9 strains from biotech and hydrocarbon containing environments), plant (2 strains), water (2 strains) and insect larvae (Supplementary Table S1).

### 2.2. DNA Extraction

DNA was extracted with an in-house protocol involving mechanical and chemical lysis. Briefly, yeast cells were incubated in 5 mL YPD (Yeast extract 10 g/L, Bacto peptone 10 g/L, glucose 10 g/L) in a sterile glass tube at 28 °C with a 160 rpm agitation overnight. A 2 mL volume of cells was centrifuged, and the supernatant was removed. An equivalent volume of 300 µL of glass beads was added, as well as 200 µL of lysis buffer (Tris 10 mM pH8, EDTA 1 mM, NaCl 100 mM, Triton 2%, SDS 1%) and 200 µL phenol/chloroform/isoamyl alcohol 25:24:1. The tubes were vortexed for 3 min and centrifuged 5 min at 13,200 rpm. The supernatant was collected, added to 500 µL pure ethanol, and mixed gently. After brief centrifugation, the DNA pellet was washed with 70% ethanol, re-suspended with Tris-EDTA, and kept at −4 °C. One microliter of RNase A (10 mg/mL) was added to the samples and incubated in a water bath at 37 °C for 15 min.

### 2.3. Genome Sequencing

The first set of 45 genomes was sequenced using a shotgun approach with the Illumina Solexa technology (paired-end 2 × 100 bp in HiSeq2000). The last 11 genomes were sequenced using a shotgun approach in a HiSeq3000 system (paired-end 2 × 150 bp) (Supplementary Table S2).

Read sequences were submitted to the European Nucleotide Archive (ENA) under BioProject accession number PRJEB42834, except for strain H222, for which reads had previously been deposited under accession number PRJEB28424 [14].

We initially intended to use the genome of strain E150 sequenced in 2004 by Génolevures as a reference (Dujon et al., 2004) [9]. However, by comparison with other *Y. lipolytica* available genomes, it appears that a quite large region was missing at the 3′ extremity of chromosome Yali0D. Thus, the Sanger reads produced by the Génoscope in 2004 were manually re-assembled in that part of the genome, and a 28,097 bp extension was added, which contains 12 protein-coding genes. The revised version of the genome sequence and annotation is provided at https://doi.org/10.57745/UZFAEN (accessed on 15 December 2022). In total, the E150 genome contains 6509 protein-coding genes, not including the 30 alternative splicing isoforms nor the 63 transposable elements with a coding sequence.

### 2.4. Bioinformatics

The raw reads were trimmed with Trimmomatic version 0.32 [30]. The clean reads were mapped with BWA version 0.7.17 [31] on the revised sequence of strain E150 (Supplementary Table S2). We used Genome Analysis Toolkit (GATK) v4.1.5.0 for variant calling and hard filtering according to GATK’s best practice [32]. This genotyping pipeline resulted in a VCF file of 196,619 biallelic variants containing 166,407 SNPs and 30,212 indels discovered across the 56 samples. The set of 166,407 biallelic SNPs was further filtered by removing SNPs with missing genotypes above 0.10 and minimum alternate allele frequency (MAF) below 0.03 using PLINK [33] version v1.90b6.16. The resulting filtered data set contained 133,528 SNP positions. 

For phylogenetic analysis, the SNP file was converted to sequences in Fasta format using a custom Perl script. A phylogenetic tree was computed with RAxML version 8.2.11 [34], performing a complete analysis (ML search + 100 bootstrapping) using the GTRGAMMA evolution model. For the phylogeny of the 32-kb heterozygous region, we used IQ-TREE version 1.6.1 and ModelFinder for the selection of the best model [35].

Population genomics analyses (π, Dxy, Fst) were performed using PopGenome package version 2.7.5 [36] on a windowed basis (window size = 10 kb). Linkage disequilibrium (LD) decay analysis was performed using vcftools v0.1.16 [37] with the --geno-r2 and --ld-widow-bp 10,000 parameters. Values were averaged when SNPs had the same distance. Ts/Tv ratio and variant impacts (classified as low, moderate, and high) were obtained using SnpEff version 5.0 [38]. For the analysis of read mapping depth, the value for each genome position was divided by the genome-wide mean depth, and then the value was log base2-transformed. Plots were obtained using the shifted average method (window: 5000; step: 500) using R.

For the pan-genome analysis, reads of A101 and H222 that did not map against the E150 genome or ribosomal [39] and mitochondrial [14] sequences were assembled with SPAdes version 3.13.1 with default parameters [40]. Resulting scaffolds over 500 bp were used to map the unmapped reads of the 54 other strains (Supplementary Table S3). Similarly, the 54 sets of unmapped reads against the A101 and H222 scaffolds were assembled and filtered at 500 bp. Only putative coding sequences were considered.

Analysis of transposable elements was performed as follows. Trimmed reads were mapped with BWA on a set of TE nucleotide sequences (Supplementary Data S1). Coverage over TE was extracted using bedtools genomecov, and subsequent analyses of TE abundance were obtained using custom R scripts.

### 2.5. Growth Conditions and Phenotyping

For the drop tests, agar media were prepared with the following carbon sources: 6 sugars (glucose, D-arabinose, L-arabinose, D-galactose, D-ribose, D-sorbose), 6 organic acids (DL-lactate, malonic acid, gluconic acid, DL-malic acid, L-malic acid, citric acid), 5 polyols (Meso-erythritol, D-arabitol, D-mannitol, xylitol, glycerol), 3 hydrophobic substrates (oleic acid, tributyrin, hexadecane), triethanolamine and ethanol (Supplementary Table S4). Carbon sources were added at a 1% final concentration to the base medium YNB (Yeast nitrogen base 1.7 g/L, Phosphate buffer 50 mM, NH_4_Cl 0.67 g/L, agar 14 g/L). Lipid media were prepared with both medium bases YNB and YP (Yeast extract 10 g/L, Bacto peptone 10 g/L, agar 12 g/L). Additional YP-based plates were prepared with 10 g/L glucose (YPD) and either different SDS concentrations (0.01%, 0.02%, and 0.03%), hygromycin 200 mg/L, or NaCl 4%. YNB plates with 1% glucose were also complemented with 9% NaCl. For the detection of protease activity, a skimmed milk agar medium was prepared as previously described [41]. Plates were incubated at 28 °C, except for YPD plates, which were incubated at 28 °C and 37 °C.

Strains were pre-cultivated overnight at 28 °C under 180 rpm agitation in 24-well microplates with 1 mL liquid YPD or 3% YNB-Glucose, depending on the medium subsequently used (YNB or YP-based media). Then, absorbance at 600 nm was measured with an LKB-Novaspec II spectrophotometer (Pharmacia). About 3.10^7^ cells were collected, centrifuged for 3 min at 5000 rpm, and suspended in 500 µL water. Then, 120 µL were transferred to a 96-well microplate, and a 96-pin replicator (Boekel Scientific) was used to inoculate the agar plates. Pictures were taken after 24 h, 48 h, 72 h, and 6 days of growth. 

Pictures were analyzed with the R package gitter version 1.1.3 with parameter contrast = 5 [42]. Gitter finds the grid of colonies from a preprocessed image and calculates the size of each colony expressed as a number of pixels. 

Differences in the sensitivity of *Y. lipolytica* strains to *Debaryomyces hansenii* killer toxins were assessed according to the method described by Woods and Bevan [43] with small modifications [44]. Killer strains (*D. hansenii* AII4bS and AII4bR) were streaked in thick lines onto the surface of YPD-MB agar (yeast extract 10 g/L, peptone 20 g/L, glucose 20 g/L, NaCl 40 g/L, methylene blue 0.6 g/L in citrate-phosphate buffer pH 4.6) previously inoculated with *Y. lipolytica* cells (10^5^ cells/plate). Cultures were incubated at 14 °C for 72 h. *Y. lipolytica* strains were considered to be sensitive to killer toxins if a clear halo was observed around the lines of *D. hansenii*. The size of the clear zone of dead cells was used as a semi-quantitative measure of sensitivity.

### 2.6. Fluorescence Microscopy

100 µL of cells were stained at room temperature by 15 min incubation with BODIPY^®^ 493/503 (Invitrogen, ThermoFisher Scientific, Villebon-sur-Yvette, France) at 1 μg/mL final concentration. Images were acquired using a Zeiss Axio Imager M2 microscope (Zeiss, Le Pecq, France), with a 100× oil immersion objective and the Zeiss fluorescence microscopy filter set 45. AxioVision 4.8 software (Zeiss, Le Pecq, France) was used for observing and recording images of stained cells.

### 2.7. Lipid Content Analysis

Two media were used to investigate lipid storage and lipid synthesis capacities. The medium for lipid accumulation was made with a limiting nitrogen concentration and excess carbon with a supply of lipids (glucose 0.5% and oleic acid 2.5%). The molar ratio of carbon over nitrogen (C/N) was 60. Therefore, 1.175 g/L NH_4_Cl was added. Similarly, the C/N ratio used for the medium for lipid synthesis was 60, and glucose 3% was the only source of carbon. Yeast cells were pre-cultivated overnight at 28 °C under 180 rpm agitation in 24-well microplates with 1 mL liquid YPD. Then, absorbance at 600 nm was measured with an LKB-Novaspec II spectrophotometer (Pharmacia). Required cell quantities (optical density of inoculation around 0.5) were centrifuged for 3 min at 5000 rpm, and pellets were suspended in a 500 µL medium for either lipid synthesis or accumulation. Samples were incubated in glass tubes with 7 mL of the medium at 28 °C with 180 rpm agitation. After 66 h of growth, all samples on the oleic acid media were centrifuged and washed three times with BSA 0.5% and once with 0.9% NaCl. Cell pellets were suspended in 1 mL water and conserved at −20 °C before lyophilization for 24 h at −55 °C (Alpha 1-2Dplus, Bioblock Scientific). After 72 h growth, samples in glucose medium were centrifuged and only washed one time with NaCl 0.9% before re-suspension in 1 mL water, conservation at −20 °C, and lyophilized. 

Lipid extraction with Gas Chromatography was performed as previously described (Morin et al., Yeast 2019; Thomas et al., 2019). Fatty acid methyl esters (FAMES) were recovered from 10 to 20 mg aliquots of freeze-dried cells using the hot methanol–H_2_SO_4_ method adapted from Browse et al. [45]. Analysis was performed through gas chromatography on a Varian 430 equipped with a flame-ionization detector and a FactorFour vf-23 ms column. FAMES were identified by comparison to commercial standards (FAME32; Supelco Sigma Aldrich, Saint-Quentin Fallavier, France) and quantified using an internal fatty acid standard (50 μg C12:0 from Sigma-Aldrich) that was added prior to transesterification. Lipid content was expressed with respect to dry-cell weight (DCW): lipid content of 1% DCW = 10 mg fatty acids per g of dry cells.

### 2.8. Statistical Analysis

All statistical analyses were performed using R [46]. For each phenotype, a one-way ANOVA was performed to test the clade effect. In a few cases, a Kruskal-Wallis test was performed because the data did not meet the assumptions of an ANOVA. When Kruskal-Wallis or ANOVA showed significant results, a Tukey’s HSD test for multiple comparisons was performed. Principal component analysis was performed on the phenotypic data using R and the FactoMineR package [47]. The genome-wide association study (GWAS) was conducted using R and the statgenGWAS package v1.0.8 [48].

## 3. Results

### 3.1. Nucleotide Diversity, Phylogeny and Population Structure

To investigate the genetic diversity of *Y. lipolytica,* we constituted a collection of 56 strains from various substrates and geographical origins. The strains from 22 countries could be grouped into eight categories of substrates (animal, biotech, food, human, hydrocarbon, soil, plant, and water). We sequenced their genomes and aligned the reads against reference genomes. Cleaned reads were first mapped against the mitochondrial genome of strain H222 [14]. The percentage of mitochondrial reads ranged from 0.2% (strain 1E07) to 15.9% (DBVPG 3374) (Supplementary Table S2). To validate strain ploidy, the reads were mapped against the *MAT* locus of W29 (*MATA* allele) and E150 (*MATB* allele). The 56 strains were effectively found to be haploids, including 33 strains with a MatA genotype and 23 with a MatB (Supplementary Table S1). The deviation from the 1:1 mating-type ratio expected under regular sexual reproduction was more marked in certain clades, which are defined below (Supplementary Table S1).

Finally, the reads were mapped to the E150 nuclear genome (Dujon et al., 2004) [9]. Overall, 196,619 variants were identified, including 166,407 SNPs, 13,791 insertions, and 16,421 deletions. We observed 37,946 singletons (variants observed in only one genome), including 30,789 SNPs and 7157 indels. Rare variants with a minor allele frequency (MAF) of 0.05 or lower accounted for 27% of all variants. The mean number of variants (SNPs and indels) per strain was 47,941, with a minimal number of 23,935 (strain W29) and a maximal one of 53,887 (strain CBS 5570). The ratio Ts/Tv between transition and transversion was homogeneous among strains, with an average value of 1.62. Variant impacts were classified by SnpEff as low, moderate, and high. Low-impact variants ranged across strains from 3418 to 8417, moderate from 1736 to 4279, and high from 131 to 267.

**Figure 1:**
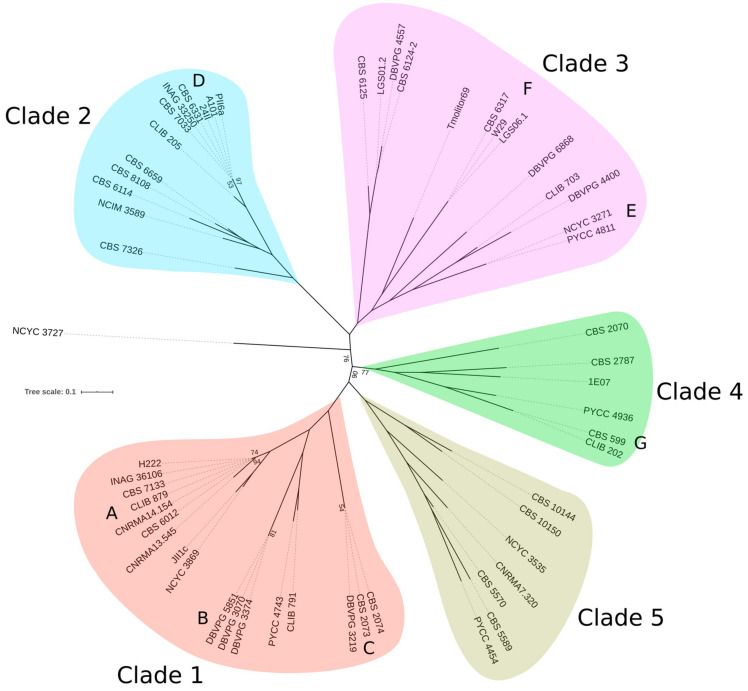
Maximum likelihood phylogenetic tree obtained from 133,528 biallelic SNPs present in at least two strains using RAxML (evolution model: GTRGAMMA). Only bootstrap values under 100 are indicated.

The genetic relationships between strains were first investigated through a phylogenetic tree based on 133,528 biallelic SNPs present in at least two strains (MAF of 0.03 or greater; maximum missing genotype below 10%). Based on this tree, five distinct clades were defined (Figure 1). We searched for a correlation between membership in a clade and geographical or ecological origin. No such population structure could be observed, as shown in Supplementary Figure S1. 

The overall nucleotide diversity π, defined as the average number of pairwise differences among sequences within the species, was low (π = 0.00177). The absolute divergence Dxy between clades was of the same order as species diversity; it reached 0.0021 between clades 2 and 4. Strains from clade 2 showed the lowest genetic diversity (π = 0.00078) by a factor of two compared to the other clades (Supplementary Table S2). The fixation index Fst varied between 0.2027 and 0.4789, suggesting that some of the clades were differentiated. Linkage disequilibrium (LD) decay with the physical distance between two SNPs showed that the half-decay distance (LD_1/2_), i.e., the distance at which LD is half of its maximum value, was about 50 bp for the whole population. LD_1/2_ is similar to whatever the clade is and equivalent to that of the whole population (Supplementary Figure S2).

The distribution of pairwise SNP differences showed a clear break in the slope between 725 and 2533 SNPs (Supplementary Figure S3). Interestingly, 25 strains were included in seven groups with highly conserved genotypes (<725 SNPs difference), which may correspond to clonemates. Three groups of such clonemates were observed in clade 1: group A (H222, INAG 36106, CBS 7133, CLIB 879, CNRMA14.154 and CBS 6012) with 349 to 725 SNP differences among the 133,528 SNP positions; group B (DBVPG 5851, DBVPG 3070 and DBVPG 3374) with less than 100 SNPs and group C (DBVPG 3219, CBS 2073 and CBS 2074) with about 130 differences between strains. All of these strains had a *MATA* allele. Group D from clade 2 comprised six *MATB* strains (PII6a, A101, 24II, CBS 6331, INAG 33250, and CBS 7033) with at most 388 SNPs differences. Clade 3 shows two groups of *MATA* strains: group E (NCYC 3271 and PYCC 4811) with 345 differences and group F (CBS 6317, W29, and LGS06.1) with at most 140 differences. Group G from clade 4 (CBS 599 and CLIB 202) displayed only 167 SNP differences. Although clade 5 had only seven strains, we did not observe any clonemates. Clade 3 has a lower percentage of clonemates (38%) compared to clade 1 (71%) or clade 2 (50%), which may contribute to the difference observed in LD decay.

### 3.2. Genome Variation: Segmental Duplication, Copy Number Variation of Genes

Analysis of copy number variations revealed that 194 out of 6509 protein-coding genes (excluding transposable elements) were missing in at least one strain (Supplementary Table S5). The core genome observed for the 56 studied strains comprised 6315 genes. For example, the 13.6-kb region encompassing the YALI0A01540g, YALI0A01562g, and YALI0A01602g genes was missing in 35 strains. YALI0A01540g and YALI0A01562g encode hypothetical proteins, and YALI0A01602g is annotated as a pseudogene in strain E150. Regarding the mating type locus, YALI0C07458g and YALI0C07480g encoding, respectively, mating type B proteins 1 and 2 were absent in all 33 *MATA* strains.

A search for individuals with varying mapping depths could not detect aneuploidies, but large segmental duplications (>100 kb) were observed in the genome of seven strains (Supplementary Figure S4). Strain CBS 7326 has a duplication of 407 kb of chromosome Yali0A. A 615 kb region of chromosome Yali0B was found to be duplicated in strain DBVPG 4557 and another region of 104 kb in CBS 599. About 112 kb of Yali0C was found in at least three copies of strain CLIB 202 and two copies of CBS 599. The same region was partly duplicated in PYCC 4743 (101 kb). A 638-kb region corresponding to the left arm of Yali0D was found duplicated in strain DBVPG 6868, in two copies in the first 623 kb, then in three copies in the last 15 kb. A region of 681 kb corresponding to the left arm of chromosome Yali0B was duplicated in strain Tmolitor69, as well as the right arm of chromosome Yali0C, which represents half of the chromosome and carries a centromere. A possible explanation is a chromosome fusion between the left arm of Yali0B and the right arm of Yali0C either during replication or autodiploidization. However, the mapping depth of the duplicated regions is not two but about 1.4, suggesting that the fusion event is not present in all cells may be due to instability.

We also searched for smaller duplications of at least 10 kb in the 56 genomes studied (Supplementary Table S6). The most frequent one was found on chromosome Yali0B between positions 96,305 (within YALI0B00748g) and 143,515 (within YALI0B00858g), resulting in a duplication of a 47-kb region with five genes coding for three proteins of unknown function, a protein of the mitochondrial complex I (N7BM) and a ribose-phosphate pyrophosphokinase highly similar to PRS5. This duplication was observed in seven strains in clade 2, including the six strains of group D and strain CLIB 205 that diverged before the group D branch.

Another duplicated region of 54 kb in Yali0B was found in three strains of clade 2 (24II, CBS 7326, and A101). Strains 24II and A101 belong to group D and CBS 7326 to a branch at the basis of clade 2, suggesting that either the duplication was ancestral to the clade and then lost in most of the strains or the duplication event occurred independently in CBS 7326 and group D.

**Figure 2:**
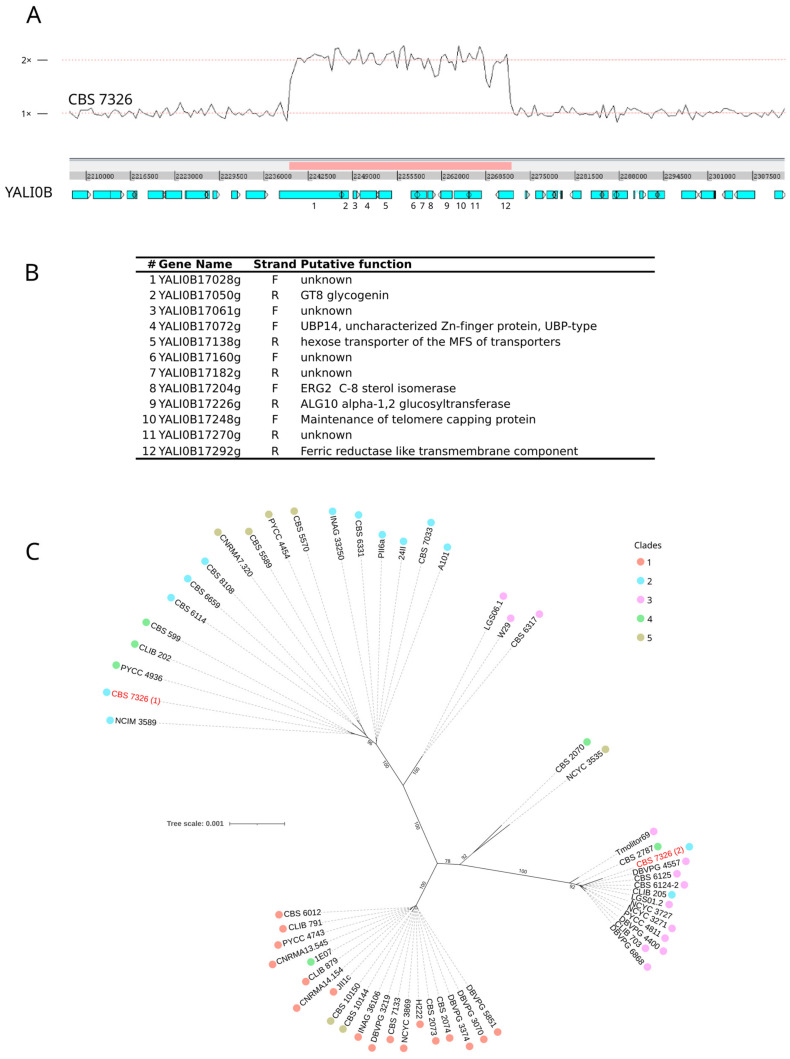
Region of 32 kb on chromosome Yali0B is duplicated in strain CBS 7326 between positions 2,239,806 and 2,271,919 of the E150 reference genome. (**A**) read mapping depth plot of the region, (**B**) list of genes present in this region, (**C**) maximum likelihood phylogenetic tree obtained using IQ-TREE (best evolution model according to BIC: HKY + F + I). The two copies of the region present in CBS 7326 are in red. Bootstrap values are indicated.

Chromosome Yali0B is duplicated between positions 2,239,806 and 2,271,919 in CBS 7326, resulting in a duplicated region of 32 kb (Figure 2A), which carries 12 genes of which five have an unknown function (Figure 2B). From the genome assembly of CBS 7326, we deduced that the duplicated copy was inserted in chromosome Yali0A between genes YALI0A02112g and YALI0A02134g. We found that this 32-kb region encompasses 77 heterozygous positions with a divergence of 2.4 per 1000 bp between the two polymorphic copies. To determine whether the duplicated copy came from a different origin or has strongly diverged, we constructed a phylogenetic tree based on this 32 kb region for the 56 studied strains, including the two-phased sequences of CBS 7326 (Figure 2C). Copy 1 clusters with strains of the same clade as CBS 7326, whereas the second copy clusters with many strains of clade 3. Surprisingly, the topology of the tree is different from that of the strain tree, suggesting possible recombination between strains of the different clades.

To detect new genes in *Y. lipolytica* genomes, we assembled sequencing reads that did not match the reference genome (E150). Only contigs larger than 500 bp were retained. In addition to the 6509 genes of E150, we found 18 new genes in H222 and A101 and an additional one in NCYC 3535, which encodes a 582 aa protein without any homolog in international databases (Supplementary Table S3, Supplementary Data S2). With this approach, the pan-genome of *Y. lipolytica* totalizes 6528 protein-coding genes, excluding transposable elements (TEs).

### 3.3. Transposable Elements

**Figure 3:**
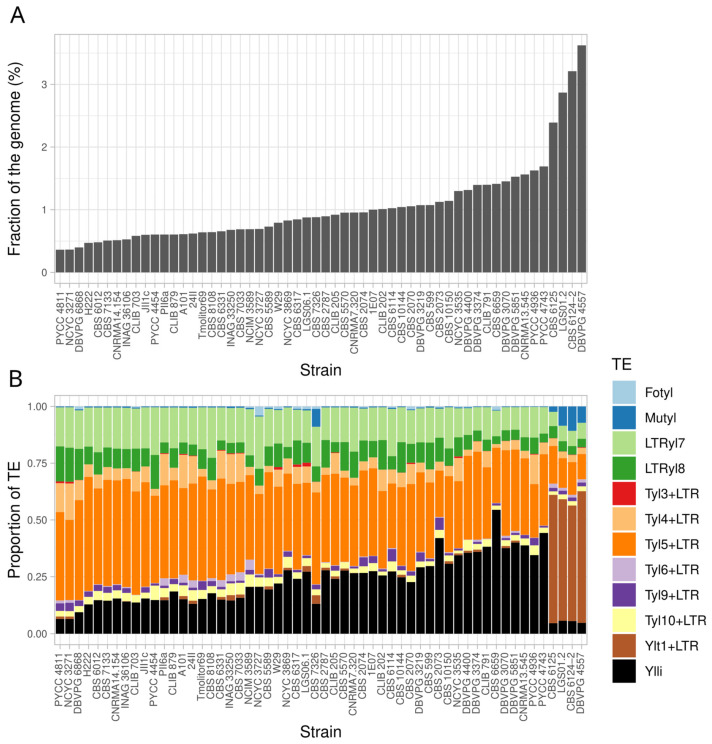
Analysis of transposable elements in the population of 56 strains. (**A**) overall amount of TEs as a fraction of the genome, (**B**) relative amount of TEs in each strain.

*Y. lipolytica* is known to carry a variety of class I and class II transposable elements [12]. For some of the LTR-retrotransposons, only solo LTRs are known. This was the case for LTRyl1 [49], which was further found full-length in strain A101 and named Tyl5 [13]. LTRyl7, LTRyl8, and LTRyl9 were found in strain W29 [12], and no full-length copy has been reported so far. By analyzing the 56 genomes, new TEs were identified, i.e., Tyl4 in CBS 599, Tyl9, and Tyl10 in PYCC 4936 (Supplementary Tables S3 and S7). Tyl9 corresponds to the full-length element of LTRyl9. Quantification of TE per genome was estimated after mapping the sequencing reads to a data set of TE sequences (Supplementary Data S1). The proportion of TEs in the genomes ranges from 0.36% to 3.62% (Figure 3A). It should be noted that four strains presented a higher quantity of TEs than the others: CBS 6125 (2.39%), LGS01.2 (2.87%), CBS 6124-2 (3.21%), and DBVPG 4557 (3.62%). This is due to the presence of a significant number of full-length Ylt1, up to 47 estimated copies in DBVPG 4557 (Figure 3B). These four strains belong to the same branch of clade 3, which suggests that only in this branch does the full-length element remain. In addition, these latter four strains and CBS 7326 from clade 2 host a complete *Mutyl* element, including *mutA* and *mutB* [20]. This Mutator-like DNA transposon is absent or at low copy numbers in the other strains, and *mutB* is missing or too degenerated to be identifiable. Overall, the most abundant TEs were the solo LTR of Tyl5 and Ylli and, to a lesser extent, LTRyl7, LTRyl8, and Tyl4 (Supplementary Figure S5). 

### 3.4. Lipid Metabolism

A list of 208 genes involved in lipid metabolism was checked for gene loss, gene duplication, and variants frequency (Supplementary Table S8). Out of the 208 genes, only YALI0A21417g was lost, which may have occurred independently in both CBS 7326 and LGS06.1 strains. Loss of this gene corresponds to the loss of subtelomeres, which includes four additional genes (YALI0A21373g to YALI0A21461g) in CBS 7326 and three in LGS06.1 (YALI0A21395g to YALI0A21461g). YALI0A21417g is a threonine aldolase with a putative hydroxytrimethyllysine aldolase function (HTMLA), i.e., the second enzyme in the carnitine biosynthesis pathway [50]. Carnitine is an important metabolite that acts as a carrier to facilitate the acetyl-CoA transfer into the mitochondria to enter the TCA cycle. Loss of the HTMLA enzyme results in dependence on exogenous carnitine.

Six genes were perfectly conserved across the 56 strains, i.e., *ALK4*, *ANT1*, *ELO1*, *HBD1*, *LIP19*, *YAS1*, and 1391 mutations were observed in the 202 other genes. These mutations had different levels of impact: 21 with high, 369 with moderate, and 1001 with low impact, according to SnpEff. Most of the 21 mutations with a high impact (stop gained, frameshift) were found in genes belonging to gene families, such as ALK or LIP genes, and therefore may have a low phenotypic impact. As expected, some mutations were shared by strains belonging to the same genetic group. For example, the last codon of *FAS1* is replaced by a stop codon in the six strains of group D. Additionally, the three Polish strains of group D have a stop codon in Arg63 of *GCY13* and a frameshift in Pro123 of *LIP17*. Interestingly, *YAS2* and *YAS3*, coding for two basic helix-loop-helix transcription activators of P450 ALK genes [51], are impacted by a frameshift (in Arg288) and a stop codon (in Leu147) in strains W29 and LGS06.1, respectively. Yas2, which forms a heterodimer with Yas1, is essential for the induction of ALK gene transcription in response to alkanes [52]. The presence of a stop codon in the first third of the mRNA probably induces nonsense-mediated mRNA decay, leading to the degradation of the transcript and preventing protein production. The mutation observed in *YAS3*, which is involved in the transcriptional repression of a variety of ALK genes [53], may also affect the assimilation of alkanes in these two strains. These findings are validated by Michely et al., who observed that W29 was not able to grow on C10, C12, and C16-alkanes unless the medium was supplemented with peptone [54].

### 3.5. Phenotypic Variation

To phenotypically characterize *Y. lipolytica,* we submitted the 56 studied strains to an analysis of 41 quantitative traits using three different methods: (i) colony size on solid media under 34 different conditions, (ii) size of a halo produced by hydrolysis of lipids (2 conditions) and skimmed milk (1 condition), or by killer toxin-induced cell death (2 killer toxins), and (iii) quantification of lipids under synthesis and accumulation media (2 conditions) (Supplementary Table S4).

Eventually, ten out of the 34 growth conditions on solid medium were at last not considered for colony size estimation. Indeed, strain growth was too weak on D- and L-arabinose, D-galactose, malonic acid, xylitol, and triethanolamine. In addition, growth on tributyrin (YNB and YP base media), oleic acid with YP base, and skimmed milk medium was not suitable for colony size quantification with the gitter package. Among the remaining 24 conditions, 15 were related to carbon source assimilation and nine to stress resistance, including two YPD controls (YPD-28C and YPD).

Regarding the assimilation of the 15 carbon sources, strains efficiently grew on all media except four, i.e., D-arabitol, D-mannitol, D-ribose, and L-sorbose. On these four media, some strains did not form any colony. The most discriminating carbon sources, i.e., those for which the coefficient of variation (CV) obtained for colony size was the highest, were D-mannitol (CV = 0.87) and L-sorbose (CV = 0.82), although growth on these media was generally weak (Supplementary Figure S6A and Supplementary Table S9). On these media, we also observed for some strains a sort of papillae, i.e., secondary colonies, suggesting an onset of spontaneous mutations in cells within the colony (Supplementary Figure S7). The less discriminating carbon sources were glycerol (CV = 0.24) and glucose (CV = 0.33), the preferred carbon source for *Y. lipolytica*. One-way ANOVA or Kruskal-Wallis tests were performed to identify a possible clade effect on colony size (Supplementary Table S10), and a statistically significant difference in colony size was observed between at least two clades on glucose, L-sorbose, DL-lactate, meso-erythritol, and hexadecane.

Stress resistance was tested on media containing NaCl, hygromycin, or SDS. Growth in the presence of an acidic pH and at 37 °C was also tested. The most discriminant stress conditions were observed when yeast cells were cultivated in the presence of hygromycin (CV = 1.74), where half of the strains were unable to grow, or when the temperature was 37 °C (CV = 1.54). In the latter condition, 28 out of 56 strains just did not grow. One-way ANOVA and Kruskal-Wallis tests statistically validated a clade effect for the following media: YPD at 37 °C, YPD with citrate (acid pH), and YPD with SDS at concentration 0.01 and 0.02% (Supplementary Figure S6B and Supplementary Table S10). By contrast, media with NaCl did not allow differentiating between clades.

**Figure 4:**
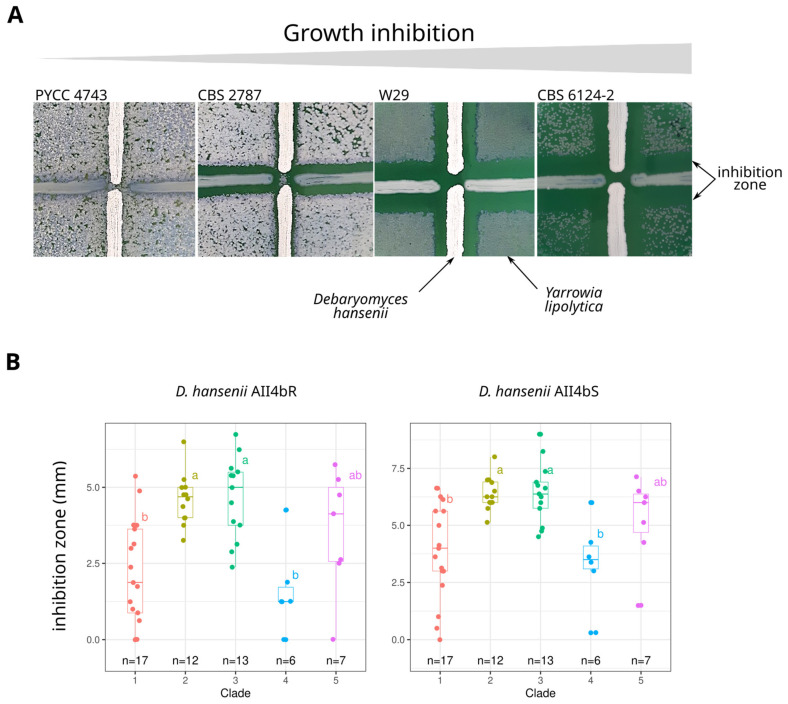
Analysis of the sensitivity to *Debaryomyces hansenii* killer toxin. (**A**) picture of the inhibition zone around *D. hansenii* cells expressing the two killer toxins (AII4bR vertical line, AII4bS horizontal line), (**B**) boxplot of the size of the inhibition zone according to the clades, each with a different colour. Letters mark significant differences between clades according to Tukey’s HSD (Honest Significant Difference) test.

Sensitivity to the killer toxin of *D. hansenii* strains AII4bR and AII4bS was estimated by measuring the inhibition zone on each side of *D. hansenii* cell lines (Figure 4A). Five strains were not sensitive to AII4bR (CBS 10150, CLIB 791, PYCC 4743, PYCC 4936, JII1c), whereas only PYCC 4743 was found resistant to AII4bS. The mean size of the inhibition zone was statistically larger with AII4bS than with AII4bR (paired samples t-test; t(55) = −24.099, *p*-value < 2.2 × 10^−16^), suggesting higher AII4bS toxicity. The one-way ANOVA tests showed a statistically significant difference in killer toxin sensitivity between at least two clades (Supplementary Table S10). Tukey’s HSD test for multiple comparisons found that the mean value of killer toxin sensitivity was significantly different between clades 2 and 3 and clades 1 and 4 (Figure 4B).

**Figure 5:**
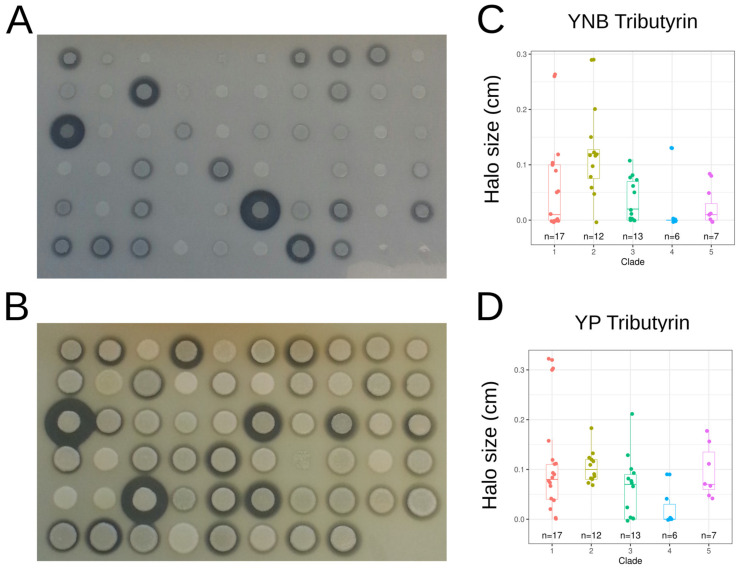
Lipase activity on tributyrin. (**A**) halo formation around colonies grown on media containing tributyrin and YNB, (**B**) halo size as a function of the clade as obtained on the YNB media, (**C**) halo formation around colonies grown on media containing tributyrin and YP, (**D**) halo size as a function of the clade as obtained on the YP media.

Extracellular lipase activity was estimated by measuring hydrolysis halo size around the colony on tributyrin media with a YNB or YP base (Figure 5A,B). This phenotypic trait was highly variable, with a CV of 1.23 and 0.81 for the media with a YNB or YP base, respectively. The largest halos on the YNB base were observed for strains CBS 6114 (0.29 cm), DBVPG 3219 (0.26 cm), CLIB 205 (0.20 cm), and INAG 33250 (0.15 cm). On the YP base medium, the mean size of the halo was larger than on the YNB base medium, the best halo-forming strains being DBVPG 3219 (0.32 cm), CBS 2073 (0.30 cm) and DBVPG 6868 (0.21 cm). A Kruskal-Wallis test showed a significant difference in halo formation between at least two clades on tributyrin media with YNB and YP base (Supplementary Table S10, Figure 5C,D).

**Figure 6:**
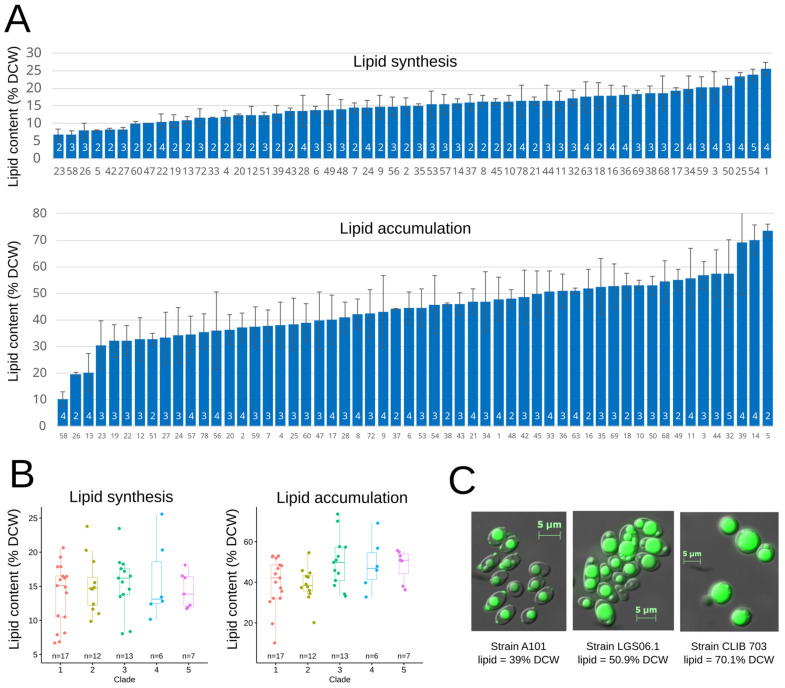
Lipid synthesis and accumulation were monitored as described in the materials and methods section. (**A**) lipid content for each strain of *Y. lipolytica*. Strain numbers in black correspond to the ID in Supplementary Table S1. The number of replicated measures is in white. (**B**) lipid content as a function of clade. (**C**) microscopic photography showing yeast cells with lipid bodies colored in green with BODIPY^®^.

Lipid synthesis of *Y. lipolytica* cells was measured after growth on glucose as the only carbon source (Figure 6A). The minimum lipid content was 6.68% (DBVPG 3374), and the maximum was 25.59% (CBS 2070). Likewise, lipid accumulation was measured after growth on a medium containing both glucose and oleic acid (Figure 6B). The minimum lipid accumulation observed was 10.10% (JII1c), with a 73.61% maximum (CBS 6125). The calculated CVs were similar for lipid synthesis (CV = 0.28) and accumulation (CV = 0.27). The top accumulating strains were not the same as the ones top performing for lipid synthesis, suggesting that lipid accumulation and synthesis are decoupled. Using one-way ANOVA, we did not observe any significant variations in lipid synthesis and accumulation among clades (Supplementary Table S10, Figure 6B). Lipid accumulation was also monitored using microscopy, enabling observation of lipid accumulation in cell lipid bodies. In the strains that accumulated the greatest amount of lipids, the lipid bodies occupied almost the entire cell (Figure 6C).

A principal component analysis (PCA) was performed on the full data set of phenotypes (Supplementary Figure S8A). On this graph, where the strains are colored according to their clade, we did not observe any grouping. Similarly, no clustering according to geographic or ecological origin could be evidenced (not shown). The amount of variance explained by the first two principal components (PC) of the PCA was only 37% (Supplementary Figure S8C). However, we could notice some correlation between certain phenotypes (Supplementary Figure S8B). There was a reverse correlation between sensitivity to both *D. hansenii* killer toxins and salt resistance (YPD with 4% NaCl). As killer toxin activity is usually measured in the presence of NaCl in the medium [55], the sensitivity of some strains to NaCl may thus have enhanced their sensitivity to the killer toxins.

### 3.6. Links between Genotypes and Phenotypes

Despite the low number of strains in the dataset, we tentatively performed a genome-wide association study on the 56 isolates and each of the 32 exploitable phenotypic traits. Some traits showed clear differences between strains, but we did not observe any associations. We, therefore, studied certain genes known to be involved in specific traits.

**Figure 7:**
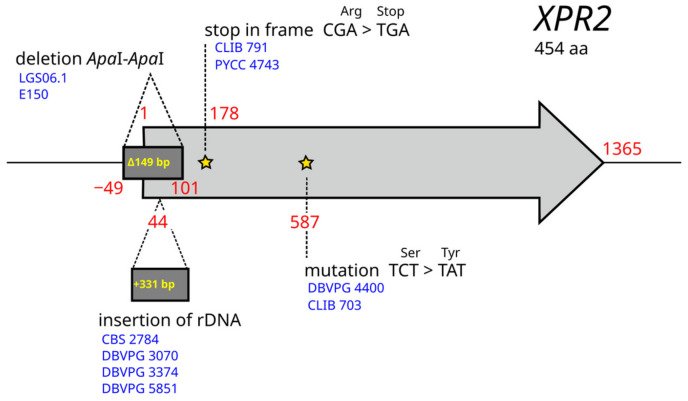
Nucleotide variations found in *XPR2*. Comparison is relative to gene YALIH222S02e00408g of *Y. lipolytica* strain H222. Variants specific to strains showing no halo on the skimmed milk medium are shown. Yellow stars indicate the nucleotide position of the mutations.

Protease activity is the sole trait that could be explained by genetic mutations. Twelve strains showed no halo on skimmed milk medium, suggesting they do not have a functional secreted protease. Since Xpr2 is the main alkaline extracellular protease of *Y. lipolytica*, we searched for mutations that occurred specifically in the 12 strains. We used YALIH222S02e00408g from strain H222 (https://gryc.inra.fr, accessed on 15 December 2022) as a reference since the *XPR2* sequence is partly deleted in E150 (Figure 7). Different genetic bases explaining the phenotype were found for nine strains. LGS06.1 shares the same 149-bp *Apa*I-*Apa*I deletion introduced in the 5′-region of *XPR2* in E150, suggesting that strain LGS06.1 is not wild [56]. A stop was observed in codon 60 (CGA to TGA) for strains CLIB 791 and PYCC 4743, which are closely related (clade 1, Figure 1). A non-synonymous mutation, Ser(TCT) to Tyr(TAT), was observed in codon 196 of *XPR2* in the closely related (clade 3) strains DBVPG 4400 and CLIB 703. The active site of Xpr2 includes three highly conserved residues, i.e., amino acids D200, H231, and S397 in YALIH222S02e00408g. Therefore, due to the proximity to codon 200, we hypothesize that the S196T mutation has an impact on Xpr2 activity. In CBS 2787, DBVPG 3070, DBVPG 3374, and DBVPG 5851, a 231-bp insertion of rDNA disrupts the 5′-region of *XPR2*. The three DBVPG strains belong to group B of clade 1, whereas CBS 2787 belongs to clade 4. As no mutation was observed in strains H222, CBS 10144, and LGS01.2, we searched for SNPs and indels with a high impact on the genome. The number of highly impacted genes was 192, 211, and 133 for H222, CBS 10144, and LGS01.2, respectively. We reduced the list of mutations to those present only in strains that did not form a halo. This provided 27 mutations in 24 genes. None of them had a function in line with protease activity, secretion, or regulation. Moreover, none of the 27 mutations were shared by the three strains. As these strains are divergent and belong to three different clades, they could have undergone different mutational events. This finding is in line with the study of Ogrydziak and Mortimer, who found 10 to 11 complementation groups for 18 mutants with reduced ability to produce a halo on skimmed-milk agar [57]. 

## 4. Discussion

Although *Yarrowia lipolytica* is a non-conventional yeast that is extensively studied, little is known about its phenotypic and genomic diversity. Here we analyzed for the first time the diversity of 56 haploid strains collected in widely diverse ecological and geographical environments. Whole genome sequencing showed a very low nucleotide diversity, with a total of 166,407 SNPs and 30,212 indels and an overall nucleotide diversity π equal to 0.00177. This diversity is more than ten times lower than that of *Brettanomyces bruxellensis* (π = 0.012 [58]) and *Lachancea kluyveri* (π = 0.017 [59]) and even lower than that of *S. cerevisiae* and *Schizosaccharomyces pombe*, both having a genetic diversity π of about 3 × 10^−3^ [60,61]. *Y. lipolytica* diversity is comparable to subclade VNIa diversity (π = 0.00178) in the VNI lineage, the less divergent lineage of *Cryptococcus neoformans* var. *grubii* [62]. Therefore, *Y. lipolytica* appears to be one of the less divergent yeast species studied so far, suggesting that it might be a recently diverging species. The fact that *Y. lipolytica* possesses a pan-genome (6528 genes), barely different from that of the core genome (6315), agrees with a recent evolution of the species.

Pairwise differences among *Y. lipolytica* strains ranged from 68 to 49,022 SNPs. A total of 25 clonemates were identified, included in seven groups with highly conserved genotypes (<725 SNPs difference between strains). Interestingly, these clonemates were isolated from very different ecological niches. The same result was observed at the clade level, suggesting that there had been no adaptive evolution to specific environments or that the set of studied strains did not allow us to detect such an ecological selection. On the contrary, it suggests that dissemination is possible between distinct environments. The most striking example is that of the Group D of clade 2 where the six clonemates were collected from cheese, soil, hydrocarbons, and a biotech environment. Moreover, these clonemates were isolated from very distant geographical places (Poland, France, the UK, and Japan), showing that dissemination may take place at very large scales, either naturally or associated with anthropic activities. 

The presence of many such clonemates suggests that propagation might be mainly clonal. Indeed, *Y. lipolytica* is known to have considerably low mating frequency and spore viability between lineages [56]. *Y. lipolytica* chromosome size polymorphism, previously reported through electrophoretic karyotype analysis [16,63] and loss of chromosome collinearity due to chromosomal rearrangements [12,14,15], may account for this poor interfertility. However, some results of our population genomics analysis show, by contrast, that clades were interbreeding populations rather than clonally propagated lineages. First, the drop in linkage disequilibrium (LD) was very steep, with a half-decay distance of only 50 bp, suggesting highly frequent recombinations. This value is extremely low compared to what had already been published: it is 10-fold lower than that obtained for *S. cerevisiae* (LD_1/2_ = 500 bp [60]) and 400-fold lower than the value reported for *S. pombe* (20 kb [61]). In addition, we observed in the haploid strain CBS 7326 a duplicated region of 32 kb showing 77 heterozygous positions. Whereas the first copy groups as expected with the homologous region of clade 2 strains, the other copy is close to those of clade 3 strains, including CBS 6124-2 (Figure 2). Moreover, the topology of the phylogenetic tree of this 32-kb region is different from the topology of the tree based on the full SNP dataset, and strains did not cluster according to the clade to which they belong. This implies that at least this region, but probably others, have been exchanged between strains. This could be generated by introgression with different consequences: duplication as in CBS 7326 or replacement of the native region by genetic conversion. 

Another unique feature of CBS 7326 in line with a putative introgression is the presence in its genome of a *Mutator*-like element called *Mutyl*, with both *mutA* and *mutB* [20]. Such full-length *Mutyl* element is exclusively present in the lineage of the American strain CBS 6124-2 (Figure 3). It was previously hypothesized that some lineages had lost *Mutyl*, which is consistent with our observation that the vast majority of strains have, at best, highly degenerated relics of *Mutyl* transposase *mutA*. Therefore, considering the origin of the second copy of the 32-kb region in CBS 7326, it can be speculated that the presence of *Mutyl* in this strain may be a re-acquisition linked to the acquisition event of the 32-kb region from a strain closely related to CBS 6124-2, CBS 6125, LGS01.2, and DBVPG 4557. Interestingly, these four strains are also unique in having full-length copies of Ylt1, an LTR-retrotransposon of the *Gypsy* family, but CBS 7326, as the majority of the 56 strains studied, did not show any presence of this TE (Supplementary Figure S5).

Overall, the TE fraction in *Y. lipolytica* genomes is quite low (from 0.36% to 3.62%), as usually reported in Saccharomycotina species [64], but the number of different elements is quite high for yeast. Two class II transposons (*Mutyl* and Fotyl) and four class I retrotransposons (Ylli, Ylt1, Tyl6, Tyl5, Tyl3) had previously been described, as well as three families of solo LTR (LTRyl7, LTRyl8, LTRyl9). Here we identified three new full-length LTR-retrotransposons (Tyl4, Tyl9, and Tyl10), all of them belonging to the *Gypsy* family [65]. In all strains, the most frequent elements are the non-LTR LINE L1 element Ylli [18] and the LTR-retrotransposon of the *Copia* family Tyl5 [13], whose cumulative proportion can reach up to 75% of the TE content. Both elements were certainly present in the ancestor of the species. All other TEs are sparse or absent and often degenerate or present as solo LTR in the case of LTR-retrotransposons. Ylt1 remains a lineage-specific exception in the four strains mentioned above as, to a lesser extent *Mutyl* in the same strains and CBS 7326. Evidencing the simultaneous presence of *Mutyl* and Ylt1 elements in *Y. lipolytica* strains is a notable result of this study. Some lineages, such as that of CBS 6124-2, appear as a reservoir of TEs. Subsequent investigations are required to understand the evolution and dynamics of these genetic elements in *Y. lipolytica* and their impact on the host genomes.

TEs are known to contribute to chromosome architecture plasticity. In a significant number of strains, we identified segmental duplications of different sizes, but we were unable to link these events to the presence of TEs. Whereas small duplications probably have a limited impact on chromosome organization, large duplications, such as those observed in CBS 7326 (406 kb), Tmolitor69 (681 kb + 1680 kb), DBVPG 4557 (615 kb), and DBVPG 6868 (623 kb), may constitute an obstacle to sexual reproduction as mentioned above. A noticeable consequence of segmental duplications is the duplication of protein-coding genes and, consequently, an expected increase in their expression level. Although 892 genes altogether are involved in these duplications, we did not observe any particular phenotypic change in line with such copy number variation.

We analyzed 41 phenotypic traits of the 56 strains by estimating colony size, halo size, killer toxin sensitivity, or lipid content. As expected, strain growth was very weak on several media, and the preferred carbon sources were glucose, glycerol, and hydrophobic substrates (tributyrin, oleic acid, and hexadecane). For all of these media, growth diversity was very low. By contrast, the highest diversity was observed on D-mannitol (CV = 0.87) and L-sorbose (CV = 0.82) and, to a lesser extent, on D-ribose (CV = 0.62). On these three media, we could observe a variety of morphotypes, i.e., absence of colony, smooth colonies, rough or star-shaped colonies due to filamentous growth, and heterogenous colonies with papillae. These secondary colonies are thought to originate from spontaneous mutations, as described in bacteria more than 60 years ago [66]. Therefore, it appears that mutant formation may be frequent in *Y. lipolytica,* which is an asset for adaptive laboratory evolution (ALE). This has been the subject of numerous studies since 2015 when a floated cell strategy was employed to enhance lipogenesis [67]. ALE was generally used to enhance strain oleogenic capacities [67,68] and their production of carboxylic acids [69] or to improve tolerance to various toxic compounds [70,71,72,73,74] and high temperatures [75]. In our study, it appears that growth at 37 °C was a highly divergent trait. At this temperature, only about one-fourth of the strains were able to grow effectively on YPD, yet no correlation could be established with strains isolated from the human body, as in the case of strains resistant to the antibiotic hygromycin.

The most striking result of phenotyping concerns lipid content stored in cells. Lipid contents resulting from de novo synthesis or from direct uptake and accumulation showed impressive diversity. Lipid synthesis in the presence of glucose resulted in lipid content varying from 6.7 to 25.6% of dry cell weight (DCW). Huge variations in lipid content had previously been reported, ranging from 3% to 45% for strains cultivated in glucose as the sole carbon source at 60 g/L [26]. Interestingly, whereas the authors report 3% lipid content for strain CBS 6303, we found a 15.6% value for the same strain (CBS 6303 = CLIB 703). This clearly shows that growth conditions, and in particular glucose concentration, are decisive for lipid synthesis [76]. Similarly, for lipid accumulation, i.e., with oleic acid and glucose as carbon sources, we observed a huge variation with lipid content reaching up to 73.6% DCW in strain CBS 6125, a strain isolated from a maize-processing plant. This level of lipid content had never been reported so far for a wild strain; *Y. lipolytica* is known to usually store around 40–45% of its DCW at most [2]. One exception is the study of Bati et al., who reported a 70.7% lipid content in strain NRRL Y-1094 grown on 18 g/L corn oil [77]. The values obtained here were confirmed by microscopic observations. Sometimes, the lipid content was so high that cells were broken between the microscope slide and coverslip (data not shown). We can hypothesize that growth conditions were optimal for lipid accumulation, i.e., a high initial inoculum, an initial C/N ratio of 60, and a low oxygenation in glass tubes. However, all strains were not able to accumulate such high amounts of lipids, even in these conditions. For example, the Polish strain JII1c, isolated from cheese, did not synthesize many lipids on glucose (6.9% of its DCW) nor accumulate them in the oleic acid medium (10.1% of its DCW). Strain JII1c may thus suffer from a deregulation of its lipid metabolism. We aimed to find genetic bases with GWAS and investigated the mutations observed in genes involved in lipid metabolism. Due to the low number of sequenced strains, GWAS did not provide any strong association. Additionally, SNPs and indels in lipid metabolic genes were not predicted to have high impacts on proteins. These results emphasize the complexity of the traits studied and suggest that the metabolic pathways leading to lipid storage might be diversely regulated. As previously reported, links between genotype and phenotype were not obvious [28] and deserve to be further investigated. 

## 5. Conclusions

In conclusion, this study provides new data about *Y. lipolytica* diversity from a phenotypic and genomic point of view. Here, we showed that nucleotide diversity in strains collected in various environments and countries is the lowest reported so far for a yeast species. In line with this low diversity, we observed a pan-genome barely different from the core genome. *Y. lipolytica* displays a huge phenotypic variation for the studied traits. Additional traits, such as the production of various compounds (polyols, organic acids, etc.) or enzymes, resistance to other stresses, and dimorphism deserve to be further investigated. GWAS was inconclusive, and the only deduced link between genotype and a phenotype trait, i.e., protease activity encoded by *XPR2*, emphasizes the complexity of the genetic bases that may impact traits. Additional genomic sequences and other genetic approaches are required to expand our knowledge of genome function and the evolution of this workhorse for biotechnology.

## Data Availability

The data presented in this study are available in Supplementary Materials. Read sequences were submitted to the European Nucleotide Archive (ENA) under BioProject accession number PRJEB42834.

## Supplementary Materials

The following supporting information can be downloaded at: https://www.mdpi.com/article/10.3390/jof9010076/s1, Figure S1: Phylogenetic tree with geographical and ecological origins of the strains; Figure S2: Decay of linkage disequilibrium with physical distance between two SNPs; Figure S3: Pairwise SNP differences between strains; Figure S4: Segmental duplications found in the genome of 7 yeasts; Figure S5: Normalized mapping depth over the sequence of all transposable elements detected in the genome of *Y. lipolytica*; Figure S6: Colony size measured after growth on different carbon sources and under different stress conditions; Figure S7: Colony size recorded on media containing L-sorbose and D-mannitol; Figure S8: Principal component analysis (PCA) computed using all phenotypic traits; Table S1: *Y. lipolytica* strains used in this study; Distribution of MatA and MatB alleles in *Y. lipolytica* strains grouped by clade; Table S2: Read number, sequencing depth and mapping statistics; Nucleotide diversity (pi), absolute divergence (Dxy) and fixation index (Fst); Table S3: Nuclear sequences from strain H222 and A101 absent from strain E150; Nuclear sequences absent from strains E150, A101 and H222; Table S4: List of media used for phenotyping; Table S5: List of missing genes in at least one strain; Table S6: Segmental duplications found in the genomes; Table S7: Characteristics of the transposable elements of *Yarrowia lipolytica*; Table S8: SNP impacts according to SnpEff in the lipid metabolism encoding genes; Table S9: Table of the results obtained after the phenotypic tests; Table S10: Statistical tests applied to phenotypic data; Data S1: Sequences of the transposable elements used in this study; Data S2: Sequences found in other genomes but not in the genome of strain E150.

## References

[1] Mamaev D., Zvyagilskaya R. (2021). *Yarrowia lipolytica*: A multitalented yeast species of ecological significance. FEMS Yeast Res..

[2] Salvador Lopez J.M., Vandeputte M., Van Bogaert I.N.A. (2022). Oleaginous yeasts: Time to rethink the definition?. Yeast.

[3] Lu R., Cao L., Wang K., Ledesma-Amaro R., Ji X.J. (2021). Engineering *Yarrowia lipolytica* to produce advanced biofuels: Current status and perspectives. Bioresour. Technol..

[4] Poli J.S., da Silva M.A., Siqueira E.P., Pasa V.M., Rosa C.A., Valente P. (2014). Microbial lipid produced by *Yarrowia lipolytica* QU21 using industrial waste: A potential feedstock for biodiesel production. Bioresour. Technol..

[5] Rong L., Miao L., Wang S., Wang Y., Liu S., Lu Z., Zhao B., Zhang C., Xiao D., Pushpanathan K. (2022). Engineering *Yarrowia lipolytica* to Produce Itaconic Acid From Waste Cooking Oil. Front. Bioeng. Biotechnol..

[6] Park Y.K., Ledesma-Amaro R. (2022). What makes *Yarrowia lipolytica* well suited for industry?. Trends Biotechnol..

[7] Madzak C. (2021). *Yarrowia lipolytica* Strains and Their Biotechnological Applications: How Natural Biodiversity and Metabolic Engineering Could Contribute to Cell Factories Improvement. J. Fungi.

[8] Barth G., Gaillardin C. (1997). Physiology and genetics of the dimorphic fungus *Yarrowia lipolytica*. FEMS Microbiol. Rev..

[9] Dujon B., Sherman D., Fischer G., Durrens P., Casaregola S., Lafontaine I., De Montigny J., Marck C., Neuveglise C., Talla E. (2004). Genome evolution in yeasts. Nature.

[10] Mekouar M., Blanc-Lenfle I., Ozanne C., Da Silva C., Cruaud C., Wincker P., Gaillardin C., Neuveglise C. (2010). Detection and analysis of alternative splicing in *Yarrowia lipolytica* reveal structural constraints facilitating nonsense-mediated decay of intron-retaining transcripts. Genome Biol..

[11] Liu L., Alper H.S. (2014). Draft Genome Sequence of the Oleaginous Yeast *Yarrowia lipolytica* PO1f, a Commonly Used Metabolic Engineering Host. Genome Announc..

[12] Magnan C., Yu J., Chang I., Jahn E., Kanomata Y., Wu J., Zeller M., Oakes M., Baldi P., Sandmeyer S. (2016). Sequence Assembly of *Yarrowia lipolytica* Strain W29/CLIB89 Shows Transposable Element Diversity. PLoS ONE.

[13] Devillers H., Brunel F., Polomska X., Sarilar V., Lazar Z., Robak M., Neuveglise C. (2016). Draft Genome Sequence of *Yarrowia lipolytica* Strain A-101 Isolated from Polluted Soil in Poland. Genome Announc..

[14] Devillers H., Neuveglise C. (2019). Genome Sequence of the Oleaginous Yeast *Yarrowia lipolytica* H222. Microbiol. Resour. Announc..

[15] Luttermann T., Ruckert C., Wibberg D., Busche T., Schwarzhans J.P., Friehs K., Kalinowski J. (2021). Establishment of a near-contiguous genome sequence of the citric acid producing yeast *Yarrowia lipolytica* DSM 3286 with resolution of rDNA clusters and telomeres. NAR Genom. Bioinform..

[16] Casaregola S., Feynerol C., Diez M., Fournier P., Gaillardin C. (1997). Genomic organization of the yeast *Yarrowia lipolytica*. Chromosoma.

[17] Schmid-Berger N., Schmid B., Barth G. (1994). Ylt1, a highly repetitive retrotransposon in the genome of the dimorphic fungus *Yarrowia lipolytica*. J. Bacteriol..

[18] Casaregola S., Neuveglise C., Bon E., Gaillardin C. (2002). Ylli, a non-LTR retrotransposon L1 family in the dimorphic yeast *Yarrowia lipolytica*. Mol. Biol. Evol..

[19] Kovalchuk A., Senam S., Mauersberger S., Barth G. (2005). Tyl6, a novel Ty3/gypsy-like retrotransposon in the genome of the dimorphic fungus *Yarrowia lipolytica*. Yeast.

[20] Neuveglise C., Chalvet F., Wincker P., Gaillardin C., Casaregola S. (2005). Mutator-like element in the yeast *Yarrowia lipolytica* displays multiple alternative splicings. Eukaryot. Cell.

[21] Fickers P., Benetti P.H., Wache Y., Marty A., Mauersberger S., Smit M.S., Nicaud J.M. (2005). Hydrophobic substrate utilisation by the yeast *Yarrowia lipolytica*, and its potential applications. FEMS Yeast Res..

[22] Meunchan M., Michely S., Devillers H., Nicaud J.M., Marty A., Neuveglise C. (2015). Comprehensive Analysis of a Yeast Lipase Family in the *Yarrowia* Clade. PLoS ONE.

[23] Takai H., Iwama R., Kobayashi S., Horiuchi H., Fukuda R., Ohta A. (2012). Construction and characterization of a *Yarrowia lipolytica* mutant lacking genes encoding cytochromes P450 subfamily 52. Fungal Genet. Biol..

[24] Haddouche R., Delessert S., Sabirova J., Neuveglise C., Poirier Y., Nicaud J.M. (2010). Roles of multiple acyl-CoA oxidases in the routing of carbon flow towards beta-oxidation and polyhydroxyalkanoate biosynthesis in *Yarrowia lipolytica*. FEMS Yeast Res..

[25] McCarthy C.G.P., Fitzpatrick D.A. (2019). Pangloss: A Tool for Pan-Genome Analysis of Microbial Eukaryotes. Genes.

[26] Carsanba E., Papanikolaou S., Fickers P., Erten H. (2020). Lipids by *Yarrowia lipolytica* Strains Cultivated on Glucose in Batch Cultures. Microorganisms.

[27] Carsanba E., Papanikolaou S., Fickers P., Erten H. (2019). Screening various *Yarrowia lipolytica* strains for citric acid production. Yeast.

[28] Hackenschmidt S., Bracharz F., Daniel R., Thurmer A., Bruder S., Kabisch J. (2019). Effects of a high-cultivation temperature on the physiology of three different *Yarrowia lipolytica* strains. FEMS Yeast Res..

[29] Walker C., Ryu S., Na H., Zane M., LaButti K., Lipzen A., Haridas S., Barry K., Grigoriev I.V., Quarterman J. (2018). Draft Genome Assemblies of Five Robust *Yarrowia lipolytica* Strains Exhibiting High Lipid Production, Pentose Sugar Utilization, and Sugar Alcohol Secretion from Undetoxified Lignocellulosic Biomass Hydrolysates. Microbiol. Resour. Announc..

[30] Bolger A.M., Lohse M., Usadel B. (2014). Trimmomatic: A flexible trimmer for Illumina sequence data. Bioinformatics.

[31] Li H., Durbin R. (2009). Fast and accurate short read alignment with Burrows-Wheeler transform. Bioinformatics.

[32] McKenna A., Hanna M., Banks E., Sivachenko A., Cibulskis K., Kernytsky A., Garimella K., Altshuler D., Gabriel S., Daly M. (2010). The Genome Analysis Toolkit: A MapReduce framework for analyzing next-generation DNA sequencing data. Genome Res..

[33] Purcell S., Neale B., Todd-Brown K., Thomas L., Ferreira M.A., Bender D., Maller J., Sklar P., de Bakker P.I., Daly M.J. (2007). PLINK: A tool set for whole-genome association and population-based linkage analyses. Am. J. Hum. Genet..

[34] Stamatakis A. (2014). RAxML version 8: A tool for phylogenetic analysis and post-analysis of large phylogenies. Bioinformatics.

[35] Nguyen L.T., Schmidt H.A., von Haeseler A., Minh B.Q. (2015). IQ-TREE: A fast and effective stochastic algorithm for estimating maximum-likelihood phylogenies. Mol. Biol. Evol..

[36] Pfeifer B., Wittelsburger U., Ramos-Onsins S.E., Lercher M.J. (2014). PopGenome: An efficient Swiss army knife for population genomic analyses in R. Mol. Biol. Evol..

[37] Danecek P., Auton A., Abecasis G., Albers C.A., Banks E., DePristo M.A., Handsaker R.E., Lunter G., Marth G.T., Sherry S.T. (2011). The variant call format and VCFtools. Bioinformatics.

[38] Cingolani P., Platts A., Wang L.L., Coon M., Nguyen T., Wang L., Land S.J., Lu X., Ruden D.M. (2012). A program for annotating and predicting the effects of single nucleotide polymorphisms, SnpEff: SNPs in the genome of *Drosophila melanogaster* strain w1118; iso-2; iso-3. Fly.

[39] Casaregola S., Neuveglise C., Lepingle A., Bon E., Feynerol C., Artiguenave F., Wincker P., Gaillardin C. (2000). Genomic exploration of the hemiascomycetous yeasts: 17. Yarrowia lipolytica. FEBS Lett..

[40] Prjibelski A., Antipov D., Meleshko D., Lapidus A., Korobeynikov A. (2020). Using SPAdes De Novo Assembler. Curr. Protoc. Bioinform..

[41] Abdelmoteleb A., Troncoso-Rojas R., Gonzalez-Soto T., González-Mendoza D. (2017). Antifungical Activity of Autochthonous *Bacillus subtilis* Isolated from *Prosopis juliflora* against Phytopathogenic Fungi. Mycobiology.

[42] Wagih O., Parts L. (2014). gitter: A robust and accurate method for quantification of colony sizes from plate images. G3.

[43] Woods D.R., Bevan E.A. (1968). Studies on the nature of the killer factor produced by *Saccharomyces cerevisiae*. J. Gen. Microbiol..

[44] Zarowska B., Wojtatowicz M., Polomska X., Juszczyk P., Chrzanowska J. (2004). Factors affecting killer activity of some yeast species occurring in Rokpol cheese. Folia Microbiol..

[45] Browse J., McCourt P.J., Somerville C.R. (1986). Fatty acid composition of leaf lipids determined after combined digestion and fatty acid methyl ester formation from fresh tissue. Anal. Biochem..

[46] R Core Team (2022). R: A Language and Environment for Statistical Computing.

[47] Lê S., Josse J., Husson F. (2008). FactoMineR: An R Package for Multivariate Analysis. J. Stat. Softw..

[48] van Rossum B., Kruijer W. statgenGWAS: Genome Wide Association Studies. https://biometris.github.io/statgenGWAS/index.html.

[49] Neuveglise C., Feldmann H., Bon E., Gaillardin C., Casaregola S. (2002). Genomic evolution of the long terminal repeat retrotransposons in hemiascomycetous yeasts. Genome Res..

[50] Strijbis K., van Roermund C.W., Hardy G.P., van den Burg J., Bloem K., de Haan J., van Vlies N., Wanders R.J., Vaz F.M., Distel B. (2009). Identification and characterization of a complete carnitine biosynthesis pathway in *Candida albicans*. FASEB J..

[51] Fukuda R. (2013). Metabolism of hydrophobic carbon sources and regulation of it in n-alkane-assimilating yeast *Yarrowia lipolytica*. Biosci. Biotechnol. Biochem..

[52] Endoh-Yamagami S., Hirakawa K., Morioka D., Fukuda R., Ohta A. (2007). Basic helix-loop-helix transcription factor heterocomplex of Yas1p and Yas2p regulates cytochrome P450 expression in response to alkanes in the yeast *Yarrowia lipolytica*. Eukaryot. Cell.

[53] Hirakawa K., Kobayashi S., Inoue T., Endoh-Yamagami S., Fukuda R., Ohta A. (2009). Yas3p, an Opi1 family transcription factor, regulates cytochrome P450 expression in response to n-alkanes in *Yarrowia lipolytica*. J. Biol. Chem..

[54] Michely S., Gaillardin C., Nicaud J.M., Neuveglise C. (2013). Comparative physiology of oleaginous species from the *Yarrowia* clade. PLoS ONE.

[55] Polomska X., Neuveglise C., Zyzak J., Zarowska B., Casaregola S., Lazar Z. (2021). New Cytoplasmic Virus-Like Elements (VLEs) in the Yeast Debaryomyces hansenii. Toxins.

[56] Barth G., Gaillardin C., Wolf W.K. (1996). Yarrowia lipolytica. Genetics, Biochemistry and Molecular Biology of Non Conventional Yeasts in Biotechnology.

[57] Ogrydziak D.M., Mortimer R.K. (1977). Genetics of Extracellular Protease Production in *Saccharomycopsis lipolytica*. Genetics.

[58] Gounot J.S., Neuveglise C., Freel K.C., Devillers H., Piskur J., Friedrich A., Schacherer J. (2020). High Complexity and Degree of Genetic Variation in Brettanomyces bruxellensis Population. Genome Biol. Evol..

[59] Friedrich A., Jung P., Reisser C., Fischer G., Schacherer J. (2015). Population genomics reveals chromosome-scale heterogeneous evolution in a protoploid yeast. Mol. Biol. Evol..

[60] Peter J., De Chiara M., Friedrich A., Yue J.X., Pflieger D., Bergstrom A., Sigwalt A., Barre B., Freel K., Llored A. (2018). Genome evolution across 1,011 *Saccharomyces cerevisiae* isolates. Nature.

[61] Jeffares D.C., Rallis C., Rieux A., Speed D., Prevorovsky M., Mourier T., Marsellach F.X., Iqbal Z., Lau W., Cheng T.M. (2015). The genomic and phenotypic diversity of *Schizosaccharomyces pombe*. Nat. Genet..

[62] Desjardins C.A., Giamberardino C., Sykes S.M., Yu C.H., Tenor J.L., Chen Y., Yang T., Jones A.M., Sun S., Haverkamp M.R. (2017). Population genomics and the evolution of virulence in the fungal pathogen *Cryptococcus neoformans*. Genome Res..

[63] Naumova E., Naumov G., Fournier P., Nguyen H.V., Gaillardin C. (1993). Chromosomal polymorphism of the yeast *Yarrowia lipolytica* and related species: Electrophoretic karyotyping and hybridization with cloned genes. Curr. Genet..

[64] Bleykasten-Grosshans C., Neuveglise C. (2011). Transposable elements in yeasts. Comptes Rendus Biol..

[65] Wicker T., Sabot F., Hua-Van A., Bennetzen J.L., Capy P., Chalhoub B., Flavell A., Leroy P., Morgante M., Panaud O. (2007). A unified classification system for eukaryotic transposable elements. Nat. Rev. Genet..

[66] Dean A.C.R., Hinshelwood C.N. (1957). The formation of papillae on bacterial colonies. Proc. R. Soc. B.

[67] Liu L., Pan A., Spofford C., Zhou N., Alper H.S. (2015). An evolutionary metabolic engineering approach for enhancing lipogenesis in *Yarrowia lipolytica*. Metab. Eng..

[68] Daskalaki A., Perdikouli N., Aggeli D., Aggelis G. (2019). Laboratory evolution strategies for improving lipid accumulation in *Yarrowia lipolytica*. Appl. Microbiol. Biotechnol..

[69] Yang X., Wang H., Li C., Lin C.S.K. (2017). Restoring of Glucose Metabolism of Engineered *Yarrowia lipolytica* for Succinic Acid Production via a Simple and Efficient Adaptive Evolution Strategy. J. Agric. Food Chem..

[70] Walker C., Ryu S., Trinh C.T. (2019). Exceptional solvent tolerance in *Yarrowia lipolytica* is enhanced by sterols. Metab. Eng..

[71] Li J., Zhu K., Miao L., Rong L., Zhao Y., Li S., Ma L., Li J., Zhang C., Xiao D. (2021). Simultaneous Improvement of Limonene Production and Tolerance in *Yarrowia lipolytica* through Tolerance Engineering and Evolutionary Engineering. ACS Synth. Biol..

[72] Wang Z., Zhou L., Lu M., Zhang Y., Perveen S., Zhou H., Wen Z., Xu Z., Jin M. (2021). Adaptive laboratory evolution of *Yarrowia lipolytica* improves ferulic acid tolerance. Appl. Microbiol. Biotechnol..

[73] Zhou L., Xu Z., Wen Z., Lu M., Wang Z., Zhang Y., Zhou H., Jin M. (2021). Combined adaptive evolution and transcriptomic profiles reveal aromatic aldehydes tolerance mechanisms in *Yarrowia lipolytica*. Bioresour. Technol..

[74] Narisetty V., Prabhu A.A., Bommareddy R.R., Cox R., Agrawal D., Misra A., Haider M.A., Bhatnagar A., Pandey A., Kumar V. (2022). Development of Hypertolerant Strain of *Yarrowia lipolytica* Accumulating Succinic Acid Using High Levels of Acetate. ACS Sustain. Chem. Eng..

[75] Qiu X., Gu Y., Du G., Zhang J., Xu P., Li J. (2021). Conferring thermotolerant phenotype to wild-type *Yarrowia lipolytica* improves cell growth and erythritol production. Biotechnol. Bioeng..

[76] Papanikolaou S., Galiotou-Panayotou M., Chevalot I., Komaitis M., Marc I., Aggelis G. (2006). Influence of glucose and saturated free-fatty acid mixtures on citric acid and lipid production by *Yarrowia lipolytica*. Curr. Microbiol..

[77] Bati N., Hammond E.G., Glatz B.A. (1984). Biomodification of fats and oils: Trials with *Candida lipolytica*. J. Am. Oil Chem. Soc..

