# Supplementary material for: Insights into the Genomic and Phenotypic Landscape of the Oleaginous Yeast Yarrowia lipolytica
Source: J Fungi (Basel). 2023 Jan 4;9(1):76. doi: 10.3390/jof9010076 (PMC9865632; doi:10.3390/jof9010076)
Supplement: Supplementary file 1 [file jof-09-00076-s001.zip › FigureS3.distance-matrix.pdf]

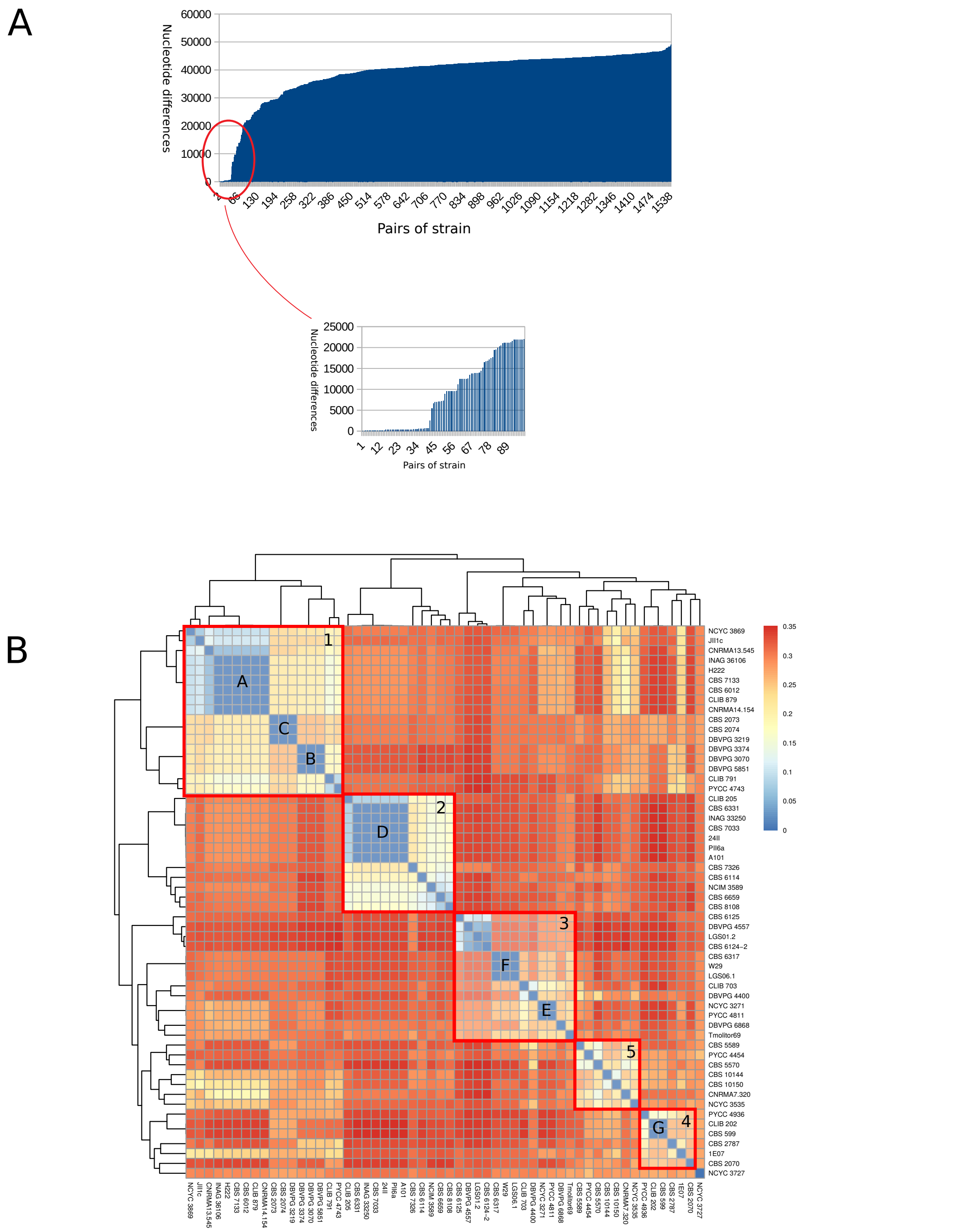

Figure S3: Pairwise SNP differences between strains.
(A) barplot showing the distribution of the pairwise difference;
(B) heatmap of the pairwise difference showing the clustering in clades and groups (red boxes). Clade numbers are the same as in Figure 1
