# Supplementary material for: Insights into the Genomic and Phenotypic Landscape of the Oleaginous Yeast Yarrowia lipolytica
Source: J Fungi (Basel). 2023 Jan 4;9(1):76. doi: 10.3390/jof9010076 (PMC9865632; doi:10.3390/jof9010076)
Supplement: Supplementary file 1 [file jof-09-00076-s001.zip › FigureS4.segmental-duplications.pdf]

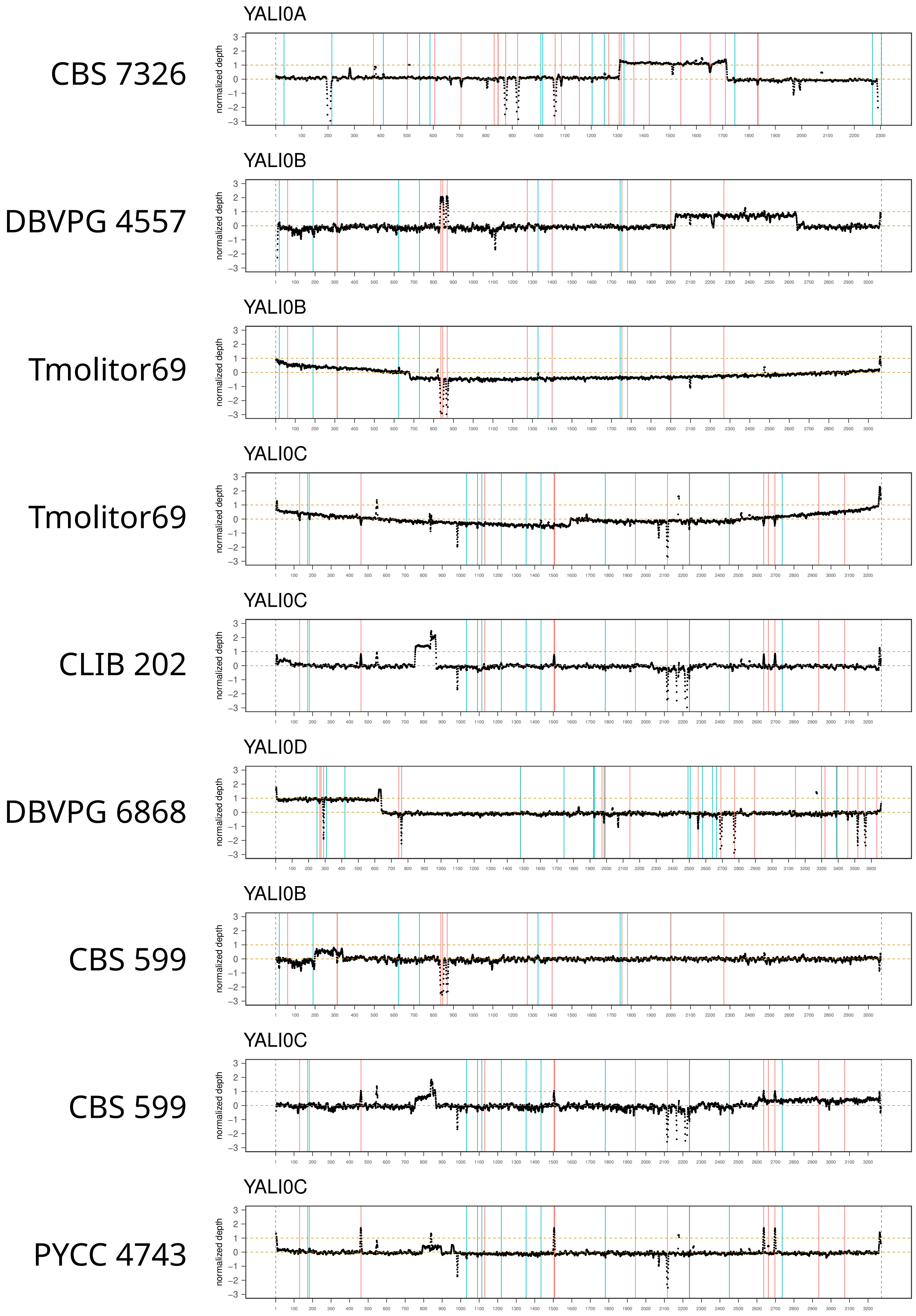

Figure S4: Segmental duplications found in the genome of 7 yeasts. Normalized mapping depth (transformed in logarithm of the base 2) was averaged over 5,000-bp windows (steps = 500 bp). Position of the transposable elements in the genome of *Y. lipolytica* E150 is marked in red and solo LTRs in blue
