# Supplementary material for: Insights into the Genomic and Phenotypic Landscape of the Oleaginous Yeast Yarrowia lipolytica
Source: J Fungi (Basel). 2023 Jan 4;9(1):76. doi: 10.3390/jof9010076 (PMC9865632; doi:10.3390/jof9010076)
Supplement: Supplementary file 1 [file jof-09-00076-s001.zip › FigureS5.TE-sequencing-depth.pdf]

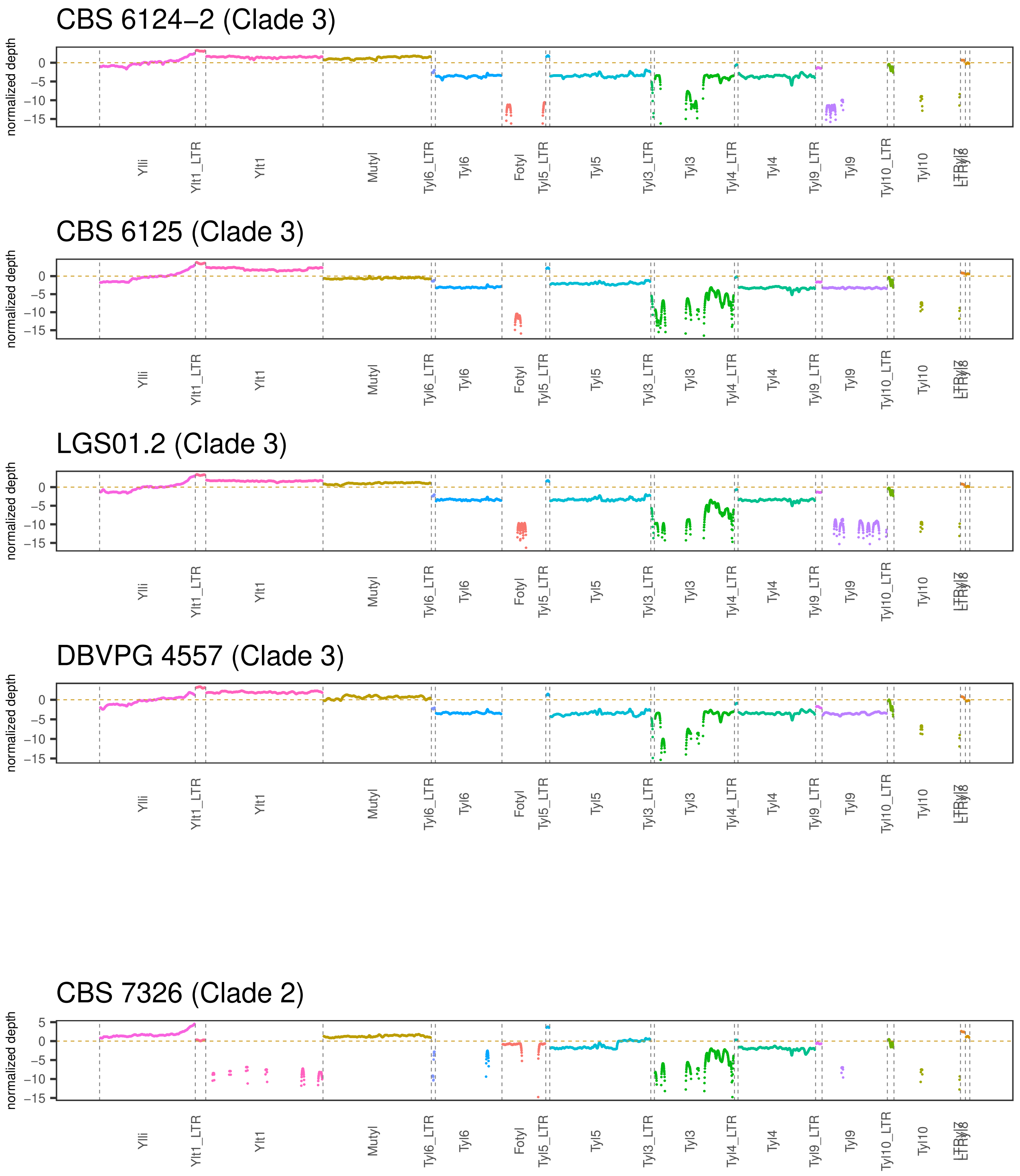

Figure S5: Normalized mapping depth (transformed in logarithm of the base 2) over the sequence of all transposable elements detected in the genome of *Y. lipolytica* strains
