# Supplementary material for: Insights into the Genomic and Phenotypic Landscape of the Oleaginous Yeast Yarrowia lipolytica
Source: J Fungi (Basel). 2023 Jan 4;9(1):76. doi: 10.3390/jof9010076 (PMC9865632; doi:10.3390/jof9010076)
Supplement: Supplementary file 1 [file jof-09-00076-s001.zip › FigureS6.phenotypes.pdf]

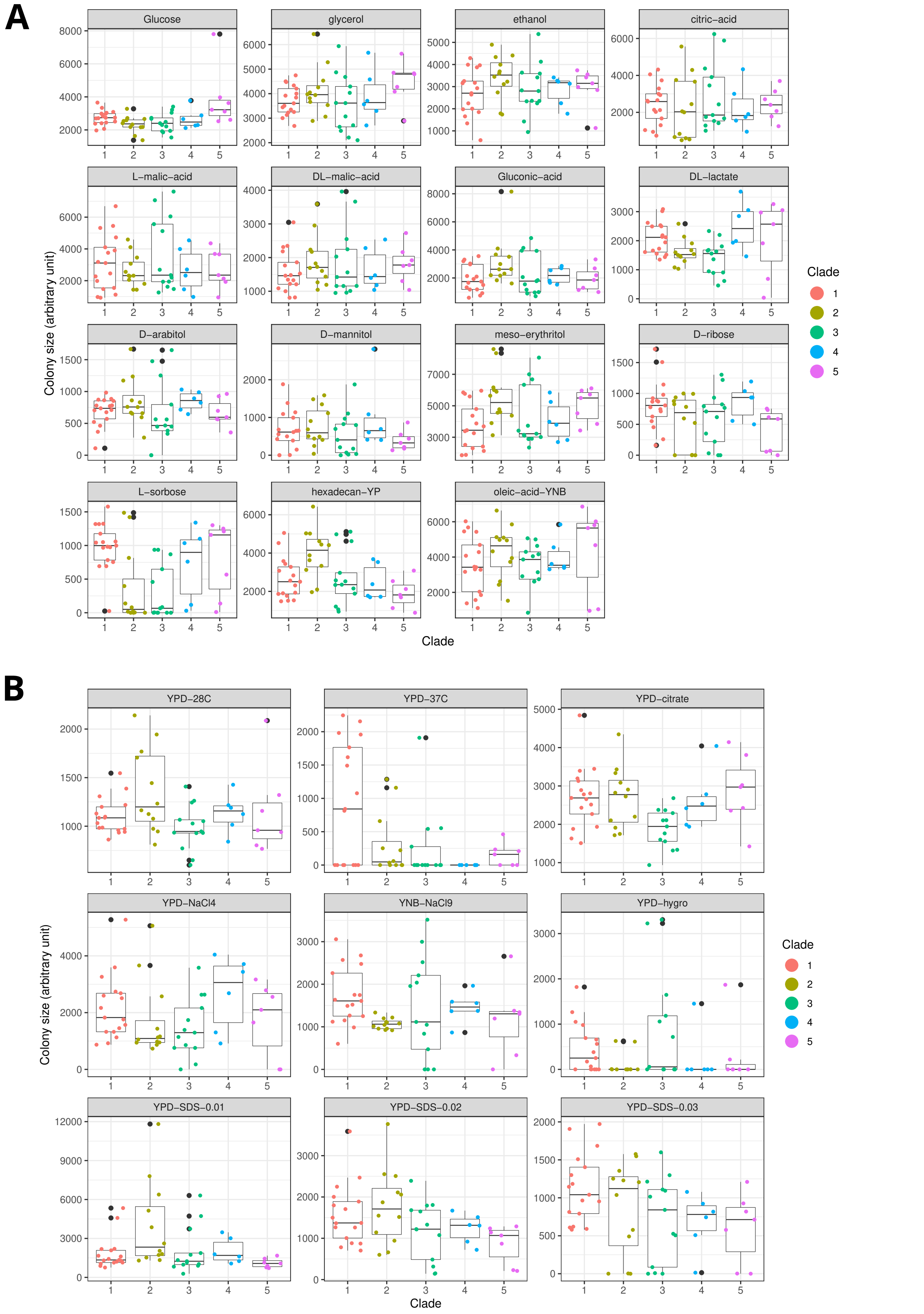

Figure S6: Colony size measured after growth on (A) different carbon sources and (B) under different stress conditions
