# Supplementary material for: Insights into the Genomic and Phenotypic Landscape of the Oleaginous Yeast Yarrowia lipolytica
Source: J Fungi (Basel). 2023 Jan 4;9(1):76. doi: 10.3390/jof9010076 (PMC9865632; doi:10.3390/jof9010076)
Supplement: Supplementary file 1 [file jof-09-00076-s001.zip › FigureS7.sorbose-mannitol.pdf]

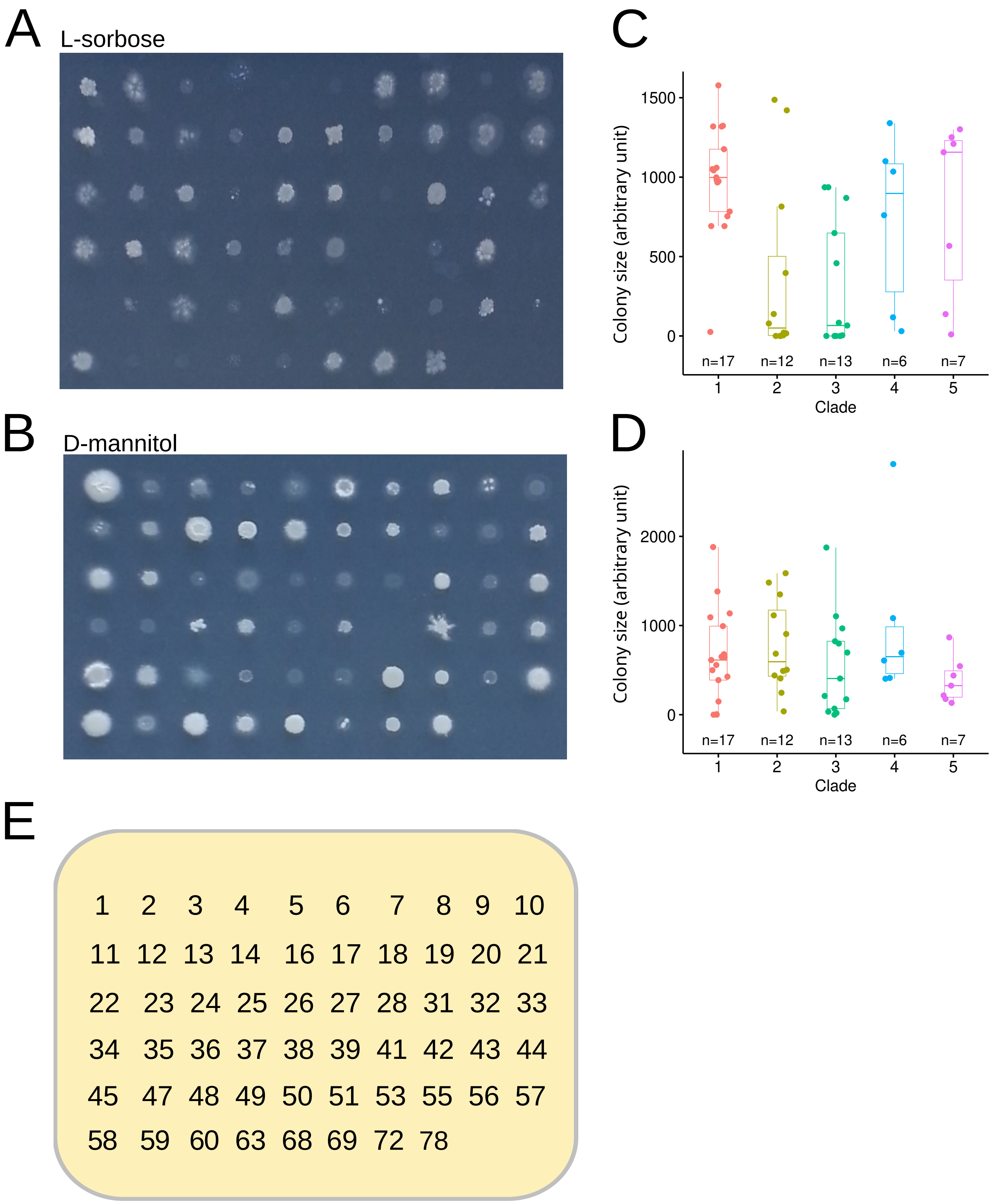

Figure S7: Colony size was recorded on media containing (A and C) L-sorbose and (B and D) D-mannitol. (E) strains were identified on the grid by their IDs (see Supplementary Table S1)
