# Supplementary material for: Insights into the Genomic and Phenotypic Landscape of the Oleaginous Yeast Yarrowia lipolytica
Source: J Fungi (Basel). 2023 Jan 4;9(1):76. doi: 10.3390/jof9010076 (PMC9865632; doi:10.3390/jof9010076)
Supplement: Supplementary file 1 [file jof-09-00076-s001.zip › FigureS8.PCA.pdf]

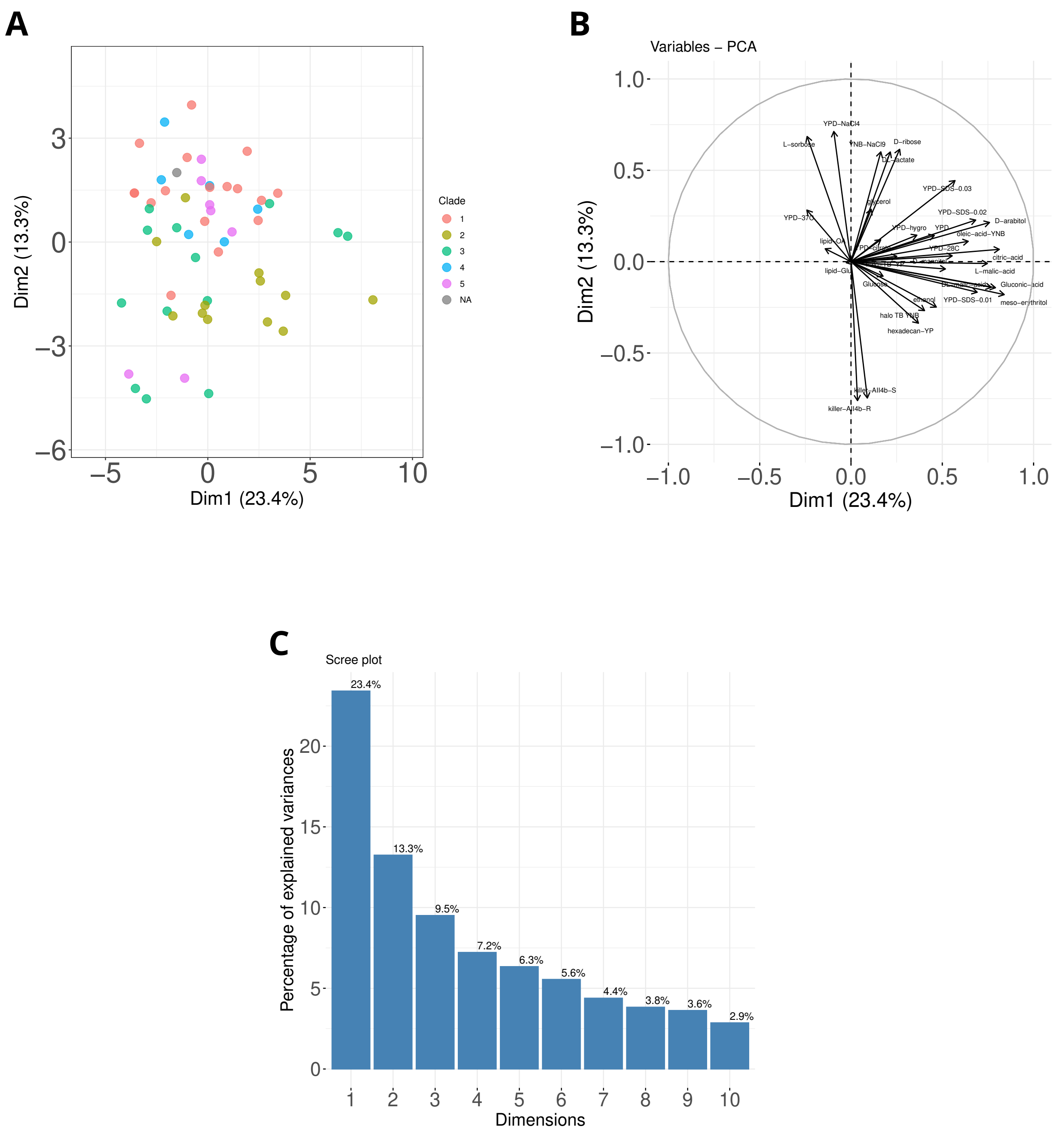

Figure S8: Principal component analysis (PCA) computed using all the phenotypic traits. (A) plot of the individuals colored according to their clade; (B) plot of the variables (traits); (C) barplot of the variance explained by each principal component
